# OLFM4 promotes intrahepatic cholangiocarcinoma progression through a PKM2-glycolysis-histone lactylation feedback loop

**DOI:** 10.7150/ijbs.135505

**Published:** 2026-09-18

**Authors:** Shuochen Liu, Kai Cao, Changan Chen, Xiao Xu, Jijun Shan, Chang Li, Ruixiang Chen, Yananlan Chen, Jiang Chang, Wangjie Jiang, Yaodong Zhang, Dong Wang, Changxian Li, Xiangcheng Li

**Affiliations:** 1 Department of Hepatobiliary Surgery, The First Affiliated Hospital with Nanjing Medical University; Key Laboratory of Liver Transplantation, Chinese Academy of Medical Sciences; NHC Key Laboratory of Living Donor Liver Transplantation, Nanjing Medical University, Nanjing, Jiangsu Province, China.; 2 The Affiliated Wuxi People's Hospital of Nanjing Medical University, Wuxi People's Hospital, Wuxi Medical Center, Nanjing Medical University, Wuxi, Jiangsu Province, China.; 3 Department of Hepatic Surgery, Fudan University Shanghai Cancer Center, Shanghai, China.; 4 Transplantation Center, The First Affiliated Hospital with Nanjing Medical University, Nanjing, Jiangsu Province, China.; 5 Jiangsu Key Laboratory of Cancer Biomarkers, Prevention and Treatment, Collaborative Innovation Center for Cancer Personalized Medicine, Nanjing Medical University, Nanjing, Jiangsu Province, China.; 6 Jiangsu Provincial Key Laboratory of Chronic Digestive Diseases, The First Affiliated Hospital with Nanjing Medical University, Nanjing, Jiangsu Province, China.

**Keywords:** OLFM4, intrahepatic cholangiocarcinoma, PKM2, glycolysis, histone lactylation

## Abstract

Metabolic reprogramming is increasingly recognized as a key characteristic of intrahepatic cholangiocarcinoma (ICC) initiation and progression. Among these metabolic alterations, glycolysis is central for supporting tumor growth. Elucidating the precise molecular mechanisms of glycolysis in ICC is critical for developing precision therapies. Here, comprehensive integrative analyses revealed that olfactomedin 4 (OLFM4) is upregulated in ICC specimens and is associated with adverse patient outcomes. Mechanistically, OLFM4 promotes ICC progression by recruiting ubiquitin specific peptidase 10 (USP10) to stabilize pyruvate kinase M2 (PKM2). This interaction enhances glycolysis and lactate production, thereby elevating histone lactylation, with a prominent impact on H3K18 lactylation (H3K18la). Consequently, H3K18la positively regulates OLFM4 transcription, establishing a positive glycolysis-histone lactylation feedback loop. Notably, tiliroside, a candidate pharmacological inhibitor of OLFM4, demonstrated antitumor efficacy in ICC. Collectively, these findings support targeting OLFM4 as a promising therapeutic strategy for ICC management.

## Introduction

Intrahepatic cholangiocarcinoma (ICC) is a highly aggressive primary liver cancer, and its global incidence and mortality continue to increase 1. Because of the silent presentation of ICC, only 20-30% of newly diagnosed patients with ICC can be offered surgical resection 2. Despite the rapid advances in diagnosis, surgical technology and systemic therapy, the long-term prognosis remains dismal, with fewer than 20% of patients surviving past five years 3. Therefore, it is crucial to identify more effective therapeutic strategies to improve patient outcomes.

Metabolic reprogramming is a fundamental mechanism of tumor progression, and ICC exhibits profound metabolic heterogeneity, influenced by multiple factors, including molecular subtypes, oncogenic drivers and microenvironmental adaptations 4. Similar to other solid tumors, ICC is highly dependent on glucose metabolism, which is a fundamental metabolic pathway in cancer and is characterized by aberrant expression of several metabolic enzymes 5, 6. Pyruvate kinase M2 (PKM2), a key enzyme in the glycolytic pathway, is frequently elevated in ICC and correlates with poor clinical outcome. Inhibition of PKM2 suppresses ICC proliferation and metastasis while increasing sensitivity to gemcitabine in ICC cells 7.

Olfactomedin 4 (OLFM4) belongs to the olfactomedin family protein and contains a conserved olfactomedin domain at the C terminus 8. Accumulating evidence indicates that OLFM4 is involved in several digestive diseases, such as inflammatory bowel disease, gallbladder cancer, colorectal cancer, and gastric cancer 9-12. Consequently, OLFM4 has emerged as a promising diagnostic indicator and a potential therapeutic target, particularly in gastrointestinal malignancies 13. However, its specific function and the molecular mechanisms by which it contributes to ICC are still unclear.

Herein, we explored the metabolic functions of OLFM4 in ICC. Our results showed that OLFM4 accelerates ICC progression by functioning as a scaffold protein that facilitates the interaction between PKM2 and the deubiquitinase ubiquitin specific peptidase 10 (USP10), thereby preventing PKM2 degradation and enhancing glycolytic activity. Furthermore, we found that the resulting lactate accumulation increases histone H3K18 lactylation, which in turn enhances OLFM4 transcription, suggesting the existence of a positive feedback loop. These findings delineate the mechanistic framework of OLFM4-driven metabolic reprogramming, highlighting its potential as a clinically exploitable target in ICC.

## Materials and Methods

Detailed materials and methods used in this study are provided in the Supplementary Materials.

## Results

### OLFM4 is overexpressed in ICC and correlates with poor prognosis

To characterize metabolic heterogeneity in ICC, consensus unsupervised clustering analysis was performed in the FU-iCCA proteomic cohort (n = 207) 14. By applying Non-negative Matrix Factorization (NMF) based on the abundance profiles of metabolic proteins represented in the human genome-scale metabolic model 15, we stratified the patients into three distinct metabolic clusters (S1-S3) (Fig. 1A, B and Fig. S1A). Compared with the S2 and S3 subtypes, patients with the S1 subtype exhibited markedly shorter overall survival (OS) (Fig. 1C). Gene Set Variation Analysis (GSVA) revealed that the S1 subtype was significantly enriched in aggressive oncogenic and inflammatory pathways, including KRAS, PI3K-AKT-mTOR, and IL6-JAK-STAT3 signaling, all of which have been implicated in ICC progression (Fig. S1B) 16, 17. To explore key targets within this aggressive subtype, we identified 51 S1-specific biomarkers (Fig. S1C). Intersecting these with upregulated genes in the GSE76297 dataset 18 yielded four candidates: TSPAN1, OLFM4, AGR2, and MMP7 (Fig. 1D, E). The prognostic value of these genes was subsequently evaluated across the FU-iCCA cohort and an independent validation cohort (GSE244807) 19. In the internal cohort, elevated expression of all four genes was associated with poor survival. In the GSE244807 dataset, OLFM4 exhibited the strongest and most robust association with poor survival (HR = 2.28, *P* < 0.001), whereas MMP7 showed no significant correlation (Fig. 1F, G and Fig. S1D, E). In addition, high OLFM4 expression was linked to advanced TNM stage, elevated CA19-9 levels, and regional lymph node metastasis (Table S1). Consequently, OLFM4 was prioritized for further mechanistic investigation.

OLFM4 was significantly upregulated in ICC samples in both the TCGA-CHOL and GSE76297 datasets (Fig. S1F, G). This increase was further verified at both the transcript and protein levels in our institutional cohort, where its high expression strongly correlated with poor survival (Fig. 1H-K). To further validate its expression pattern, single-cell RNA-sequencing and spatial transcriptomic datasets were analyzed 20, 21, confirming elevated OLFM4 expression in ICC and indicating that OLFM4 expression was predominantly localized to malignant cells and cholangiocytes (Fig. 1L, M and Fig. S1H-J).

Collectively, these findings indicate that OLFM4 is elevated in ICC and suggest its involvement in ICC progression.

### OLFM4 promotes ICC malignancy *in vitro* and* in vivo*

To elucidate the biological functions of OLFM4 in ICC, we measured OLFM4 expression in ICC cell lines (Fig. S2A, B). Then, we introduced shRNA-mediated silencing of OLFM4 in RBE cells, which exhibited comparatively high endogenous expression of OLFM4. We used lentiviral vectors to mediate OLFM4 overexpression in CCLP-1 cells, which expressed relatively low levels of OLFM4. The efficiencies were verified, and sh1 and sh3 were chosen for subsequent experiments (Fig. S2C-F).

Cell Counting Kit-8 (CCK-8), EdU, and colony formation assays showed that OLFM4 depletion suppressed ICC cell proliferation, while OLFM4 overexpression exerted the opposite effect (Fig. S3A-F). Similarly, Transwell and wound healing assays showed that OLFM4 depletion impaired ICC cell migration, whereas its upregulation enhanced migratory activity (Fig. S3G-J). Collectively, these findings suggest that OLFM4 enhances the proliferative and migratory properties of ICC cells.

To further evaluate the functional role of OLFM4 *in vivo*, RBE cells with stable OLFM4 knockdown and CCLP-1 cells with stable OLFM4 overexpression were subcutaneously injected into nude mice to establish xenograft models. In these models, OLFM4 deficiency markedly suppressed tumor growth, whereas OLFM4 overexpression accelerated tumor growth compared with the corresponding control groups (Fig. 2A-C). Immunohistochemistry (IHC) staining showed that Ki-67 levels were positively associated with OLFM4 abundance (Fig. 2D). Moreover, in the lung metastasis model, bioluminescence imaging revealed that OLFM4 knockdown markedly inhibited metastasis, whereas its overexpression promoted pulmonary colonization (Fig. 2E-H).

Furthermore, using the Sleeping Beauty system 22-25, we induced primary liver tumorigenesis via hydrodynamic tail vein injection of KRAS^G12D^/P19 CRISPR (KRAS/P19) (Fig. 2I). The results of IHC confirmed cytokeratin 19 (CK19)-positive ICC. Evaluation of the liver/body weight ratio (LBR) revealed an elevated tumor burden in the OLFM4-overexpressing group (Fig. 2J, K). Survival analysis demonstrated that OLFM4 overexpression significantly reduced overall survival in mice (Fig. 2L). Consistent findings were obtained in an additional primary ICC model driven by myr-AKT/YAP^S127A^ (YAP/AKT) (Fig. 2M-P). Overall, these data suggest that OLFM4 contributes to ICC development and progression.

### OLFM4 promotes glycolysis in ICC

Given that OLFM4 might participate in metabolic pathways, untargeted metabolomics was performed in CCLP-1 cells with OLFM4 overexpression (Fig. 3A). A total of 104 metabolites differed significantly between OLFM4-OE cells and vector cells (VIP > 1, *P* < 0.05). Kyoto Encyclopedia of Genes and Genomes (KEGG) analysis showed significant enrichment in multiple glycolysis-related pathways, such as alanine, aspartate and glutamate metabolism, one carbon pool by folate, TCA cycle, and glycine, serine and threonine metabolism (Fig. 3B). Consistently, KEGG analysis and Gene set enrichment analysis (GSEA) of the FU-iCCA cohort proteomic dataset showed that OLFM4 was positively associated with glycolysis pathway (Fig. 3C and Fig. S4A). In spatial transcriptomics 26, tumor regions with high OLFM4 expression were associated with high levels of glycolysis (Fig. 3D). Based on these findings, we hypothesized that OLFM4 promotes ICC progression by reprogramming glucose metabolism. To validate this, we measured the extracellular acidification rate (ECAR) and observed that OLFM4 overexpression significantly increased ECAR, whereas silencing OLFM4 suppressed it (Fig. 3E, F). Furthermore, OLFM4 overexpression enhanced glucose uptake and lactate production (Fig. 3G-J). To determine whether OLFM4-mediated ICC progression was dependent on glycolysis, ICC cells were exposed to the glycolytic inhibitor 2-deoxy-D-glucose (2-DG), followed by assessment of proliferative capacity using CCK-8 and colony formation assays. OLFM4-induced oncogenic growth was attenuated by 2-DG treatment (Fig. S4B, C). In short, these results suggest that OLFM4 promotes ICC progression via the acceleration of glycolysis.

### OLFM4 interacts with PKM2 to regulate glycolysis in ICC

To further elucidate the mechanism responsible for OLFM4-mediated ICC progression, immunoprecipitation-mass spectrometry (IP-MS) was performed to screen for proteins interacting with OLFM4 and the resulting candidates were integrated with upregulated proteins between OLFM4-high group and OLFM4-low group in FU-iCCA proteomics (Fig. 4A). The results showed that pyruvate kinase M2 (PKM2) was the only glycolysis-related candidate protein that interacted with OLFM4, which could be regulated by OLFM4 (Fig. 4B and Fig. S5A). Therefore, we hypothesized that OLFM4 interacts with PKM2 to regulate glycolysis in ICC.

Then, the association between OLFM4 and PKM2 was verified by endogenous Co-IP in ICC cells and exogenous Co-IP in HEK293T cells (Fig. 4C-E and Fig. S5B). Glutathione S-transferase (GST) pull-down assays demonstrated that OLFM4 directly interacts with PKM2 (Fig. 4F). Co-localization was confirmed by immunofluorescence in ICC cells (Fig. 4G).

Furthermore, to identify the domains required for the OLFM4-PKM2 interaction, a series of OLFM4 and PKM2 truncated mutants were generated for Co-IP assays (Fig. 4H). The results revealed that the C-terminal region of OLFM4 (residues 245-510) was mainly responsible for binding to the middle region of PKM2 (residues 43-388) (Fig. 4I, J).

PKM2 serves as a key regulator of glycolysis and tumor progression. To confirm whether OLFM4 regulates glycolysis and oncogenic functions through PKM2, we silenced PKM2 and evaluated ECAR, glucose uptake and lactate production in ICC cells. PKM2 depletion markedly attenuated the OLFM4-induced increases in ECAR, glucose uptake, and lactate production (Fig. S5C-E). Moreover, the OLFM4-induced increase in proliferation of ICC cells was also attenuated by PKM2 knockdown (Fig. S5F, G). These results indicate that PKM2 plays a crucial role in OLFM4-induced glycolysis and ICC progression.

### OLFM4 suppresses K48-linked ubiquitin-mediated degradation of PKM2

Proteomic analysis revealed a positive association between PKM2 and OLFM4. Western blotting demonstrated that OLFM4 overexpression elevated PKM2 protein abundance, whereas OLFM4 depletion reduced it (Fig. 5A, B). Consistently, multiplex immunohistochemistry (mIHC) analysis revealed that tumors with high OLFM4 expression also exhibited elevated PKM2 expression (Fig. 5C). Moreover, compared with OLFM4-FL, OLFM4-ΔC exhibited markedly reduced effects on ICC cell proliferation and migration (Fig. S6A, B). Consistently, full-length OLFM4 increased PKM2 protein levels, intracellular lactate levels, and glucose uptake, while these promoting effects were attenuated by deletion of the C-terminal region (Fig. S6C-E). Cycloheximide (CHX) chase assays further indicated that OLFM4 enhanced the stability of PKM2 (Fig. 5D-G). Furthermore, the reduction in PKM2 levels induced by OLFM4 knockdown was reversed by the proteasome inhibitor MG132, whereas treatment with the lysosomal inhibitor chloroquine (CQ) had no comparable effect, indicating that OLFM4 prevents PKM2 degradation in a proteasome-dependent manner (Fig. 5H, I). Consistent with this, ubiquitination of PKM2 was increased with OLFM4 knockdown and reduced by OLFM4 overexpression (Fig. 5J, K). Moreover, OLFM4 specifically regulated K48-linked ubiquitination of PKM2 (Fig. 5L-N). These data indicate that OLFM4 stabilizes PKM2 by protecting it from K48-linked ubiquitin-mediated degradation.

### OLFM4 enhances PKM2 deubiquitination by recruiting USP10

Given that OLFM4 lacks intrinsic deubiquitinase activity, we hypothesized that it functions by recruiting a specific ubiquitin-modifying enzyme to regulate PKM2 stability. Based on the IP-MS results, we identified USP10 and TRIM25 as potential candidates involved in the regulation of PKM2 ubiquitination by OLFM4 (Fig. 6A). Although both USP10 and TRIM25 interacted with OLFM4 and PKM2, only USP10 inhibited K48-linked ubiquitination and increased PKM2 protein stability (Fig. 6B-G).

Furthermore, OLFM4 overexpression enhanced the interaction between PKM2 and USP10, whereas OLFM4 knockdown suppressed it (Fig. 6H). Consistently, OLFM4 specifically facilitated USP10-induced K48-linked deubiquitination of PKM2 (Fig. 6I). To determine the specific ubiquitination site on PKM2, we constructed PKM2 mutants and found that the K206R mutation largely attenuated K48-linked ubiquitination, indicating that lysine 206 is the key target site regulated by USP10 (Fig. 6J). Functional rescue assays in PKM2-knockdown ICC cells further revealed that while OLFM4 overexpression significantly increased PKM2 protein abundance, enhanced proliferative, migratory, and glycolytic phenotypes in the presence of wild-type PKM2, these effects were substantially attenuated in cells expressing the PKM2-K206R mutant (Fig. S7A-F).

To elucidate the structural basis of this interaction, molecular docking of the OLFM4/PKM2/USP10 complex was performed. Molecular docking predicted that residues N371, Y375, N377, and E399 in the 245-510 region of OLFM4 interacted with residues R277, D280, R315, and R318 in the 43-388 region of PKM2 (Fig. 6K). To validate this prediction, the indicated interface residues were substituted with alanine. Co-IP assays confirmed that mutations in either OLFM4 or PKM2 disrupted their interaction (Fig. 6L). Crucially, mutation of the OLFM4-PKM2 binding interface attenuated OLFM4-mediated recruitment of USP10 to PKM2, consequently increasing PKM2 ubiquitination and reducing PKM2 protein abundance (Fig. 6M-O). Collectively, these findings support a scaffolding role for OLFM4 in strengthening the USP10-PKM2 interaction, thereby promoting PKM2 deubiquitination and stabilization.

### OLFM4 enhances histone lactylation and H3K18la positively regulates OLFM4 expression

Lactate, a key metabolite during glycolysis, has been reported to regulate histone lactylation. Given that OLFM4 promotes glycolytic activity and lactate accumulation, we investigated whether OLFM4 influences histone lactylation in ICC. Western blotting showed markedly elevated pan-lysine lactylation (Pan-Kla) in OLFM4-overexpression cells (Fig. 7A). Notably, among various histone lactylation modifications, H3K18la exhibited the most profound increase (Fig. 7B).

To examine the link between glycolysis and histone lactylation, ICC cells were treated with lactate or oxamate, a lactate dehydrogenase A (LDHA) inhibitor. Oxamate reduced H3K18la levels in a dose-dependent manner, whereas lactate treatment increased H3K18la levels. Interestingly, oxamate treatment also decreased OLFM4 expression, whereas lactate treatment increased OLFM4 expression (Fig. 7C, D). We next investigated whether the regulation of Pan-Kla and H3K18la by OLFM4 is dependent on PKM2 in ICC cells. Western blotting demonstrated that PKM2 knockdown attenuated the OLFM4-induced increases in Pan-Kla and H3K18la (Fig. 7E).

Given the role of histone lactylation in transcriptional regulation, genome-wide CUT&Tag profiling was performed to identify genes potentially associated with H3K18la in ICC. The results revealed significant enrichment of H3K18la peaks in the OLFM4-overexpression group relative to controls (Fig. 7F). Integrative Genomics Viewer (IGV) analysis showed marked H3K18la enrichment at the OLFM4 promoter, with increased signals at the transcription start site (Fig. 7G). ChIP-qPCR analysis further verified that H3K18la enrichment at the OLFM4 promoter was significantly elevated in OLFM4-overexpression ICC cells (Fig. 7H).

E1A binding protein p300 (P300) has been reported as a putative 'writer' of histone lactylation. Western blotting revealed that P300 expression was increased in the OLFM4-overexpression cells (Fig. 7I). Rescue experiments demonstrated that P300 silencing attenuated the OLFM4-induced increase in H3K18la (Fig. 7J). To examine whether these observations were recapitulated *in vivo*, we performed mIHC staining of tumor sections from the ICC model. Consistently, PKM2 and H3K18la levels were increased in OLFM4-overexpressing mice compared with the control group (Fig. 7K).

These results suggest that OLFM4 enhances H3K18la modification through P300 upregulation, thereby establishing a positive feedback loop that drives OLFM4 transcription in ICC.

### Tiliroside is identified as a potential OLFM4 inhibitor in ICC

Given the oncogenic role of OLFM4 in ICC, we aimed to search for potential inhibitors targeting OLFM4. However, there are no available OLFM4 inhibitors. After predicting potential binding pockets on OLFM4, we performed virtual screening using the T001 compound library. The top compounds were selected according to docking scores and MM/GBSA binding free energies. Of these, four commercially available compounds were selected for experimental validation (Fig. 8A and Fig. S8A). Among these, tiliroside exhibited the strongest inhibitory effect on ICC cell viability (Fig. 8B). Molecular docking further suggested that tiliroside could bind to the predicted pocket of OLFM4 (Fig. 8C and Fig. S8B).

To elucidate its functional impacts and determine whether they were dependent on OLFM4, RBE cells with or without stable OLFM4 knockdown were treated with tiliroside. The results showed that tiliroside reduced PKM2 protein abundance and impaired cell proliferation, migration, and glycolytic activity. However, these effects were substantially attenuated following OLFM4 knockdown (Fig. S9A-F). Together, these results indicate that tiliroside exerts its inhibitory effects on ICC cells through an OLFM4-dependent mechanism.

To further evaluate its antitumor activity in ICC *in vivo,* xenograft assays were conducted and the results showed that tiliroside markedly suppressed tumor growth (Fig. 8D-G). Serum biochemical analyses and histopathological examination of major organs revealed no apparent systemic toxicity following tiliroside treatment (Fig. S10A-D). Similarly, tiliroside significantly reduced the pulmonary metastatic burden (Fig. 8H, I).

In addition, the antitumor efficacy of tiliroside was evaluated in the KRAS/P19-driven primary ICC model. Tiliroside treatment reduced tumor burden and lowered the liver/body weight ratio (Fig. 8J, K). Survival analysis showed that tiliroside treatment significantly prolonged mouse survival (Fig. 8L). Consistent findings were obtained in the YAP/AKT model (Fig. 8M-O). Collectively, tiliroside functions as a potential pharmacological inhibitor of OLFM4 and suppresses ICC development and progression.

## Discussion

Reprogramming energy metabolism is a fundamental hallmark of cancer, enabling malignant cells to satisfy the substantial energetic requirements necessary for survival 27. ICC is a highly heterogeneous malignancy, and a deeper understanding of its metabolic diversity may pave the way to identify new treatment approaches 4. We classified ICC into three metabolic subtypes (S1-S3), each characterized by distinct metabolic patterns and clinical outcomes. The S1 subtype, which was associated with poorer survival, showed the activation of oncogenic and inflammatory signaling pathways, including KRAS, PI3K-AKT-mTOR, and IL6-JAK-STAT3 signaling. These pathways are closely linked to metabolic reprogramming and tumor aggressiveness. In contrast, the S2 subtype was enriched in Bile Acid and Fatty Acid Metabolism, suggesting a more differentiated state that correlates with better prognosis. Collectively, these findings uncover the close correlations between heterogeneity and survival differences in ICC.

Among proteins specifically upregulated in the S1 subtype, OLFM4 was prioritized for further investigation. In alignment with previous observations in other gastrointestinal cancers 12, 28, our study shows that OLFM4 is significantly upregulated in ICC tissues and promotes tumor progression and glycolysis.

Mechanistically, we demonstrated that OLFM4 functions as a scaffold to recruit the deubiquitinating enzyme USP10 to PKM2. Previous studies have shown that the stability of PKM2 can be regulated by the ubiquitin-proteasome system, involving various E3 ligases and deubiquitinases 29-31. Here we report that USP10 is a deubiquitinase of PKM2 that removes K48-linked ubiquitin chains, thereby preventing its proteasomal degradation. Importantly, OLFM4 strengthens the USP10-PKM2 association, thereby stabilizing PKM2, enhancing glycolysis and promoting tumorigenesis. Furthermore, we identified K206 as a ubiquitination site involved in this regulation of PKM2. However, because endogenous ubiquitination at K206 was not directly confirmed by mass spectrometry, the contribution of additional ubiquitination sites cannot be excluded and warrants further investigation.

Recent evidence has shown that lactate, the end product of glycolytic metabolism, exerts diverse effects on cancer biology, including regulating signaling pathways, enhancing resistance to oxidative stress, and driving lactylation 32. Lactylation connects metabolism and epigenetic regulation, such as histone lactylation regulating the transcriptional activation of genes 33. H3K18la has been implicated in oncogenesis, immune escape and metabolic reprogramming 34-36. In this study, we showed that OLFM4-driven glycolysis elevates lactate production, thereby upregulating the level of histone lactylation, with H3K18la showing the most profound increase. As a result, H3K18la promotes OLFM4 transcription and creates a positive feedback loop in ICC.

Given the critical role of OLFM4 in ICC oncogenesis, targeting it offers a promising therapeutic strategy. Structure-based virtual screening has evolved into a powerful approach for exploring potential small-molecule therapeutics 37. In the present study, we conducted virtual screening and identified tiliroside as a potential small-molecule inhibitor of OLFM4. Tiliroside, a naturally occurring flavonoid found in various edible plants and plant-derived products, has exhibited therapeutic efficacy in multiple preclinical models of tumors, inflammation and metabolism-related diseases 38-40. Our subsequent experiments showed that tiliroside inhibited ICC cell proliferation, migration, and glycolysis *in vitro* and suppressed tumor growth and metastasis *in vivo*. Moreover, these inhibitory effects were substantially attenuated following OLFM4 knockdown, supporting an OLFM4-dependent mechanism. Nevertheless, as a natural product, tiliroside may act on multiple molecular targets, and potential off-target effects cannot be excluded. Therefore, tiliroside should currently be regarded as a potential pharmacological inhibitor of OLFM4. Further studies are required to confirm its target selectivity, while additional pharmacokinetic evaluation and optimization will be necessary before clinical translation.

Several limitations of the present study should be considered. First, larger multi-center cohorts are required to further validate our findings. Second, given the importance of immunotherapy in current cancer treatment, the interplay between OLFM4 and the tumor immune microenvironment warrants further investigation.

In conclusion, our findings show that OLFM4 promotes tumorigenesis in ICC by functioning as a scaffold that recruits USP10 to deubiquitinate and stabilize PKM2, thereby reinforcing a glycolysis-H3K18la positive feedback loop. These results highlight OLFM4 as a promising therapeutic target for ICC.

## Supplementary Material

Supplementary materials and methods, figures and tables.

## Figures and Tables

**Figure 1:**
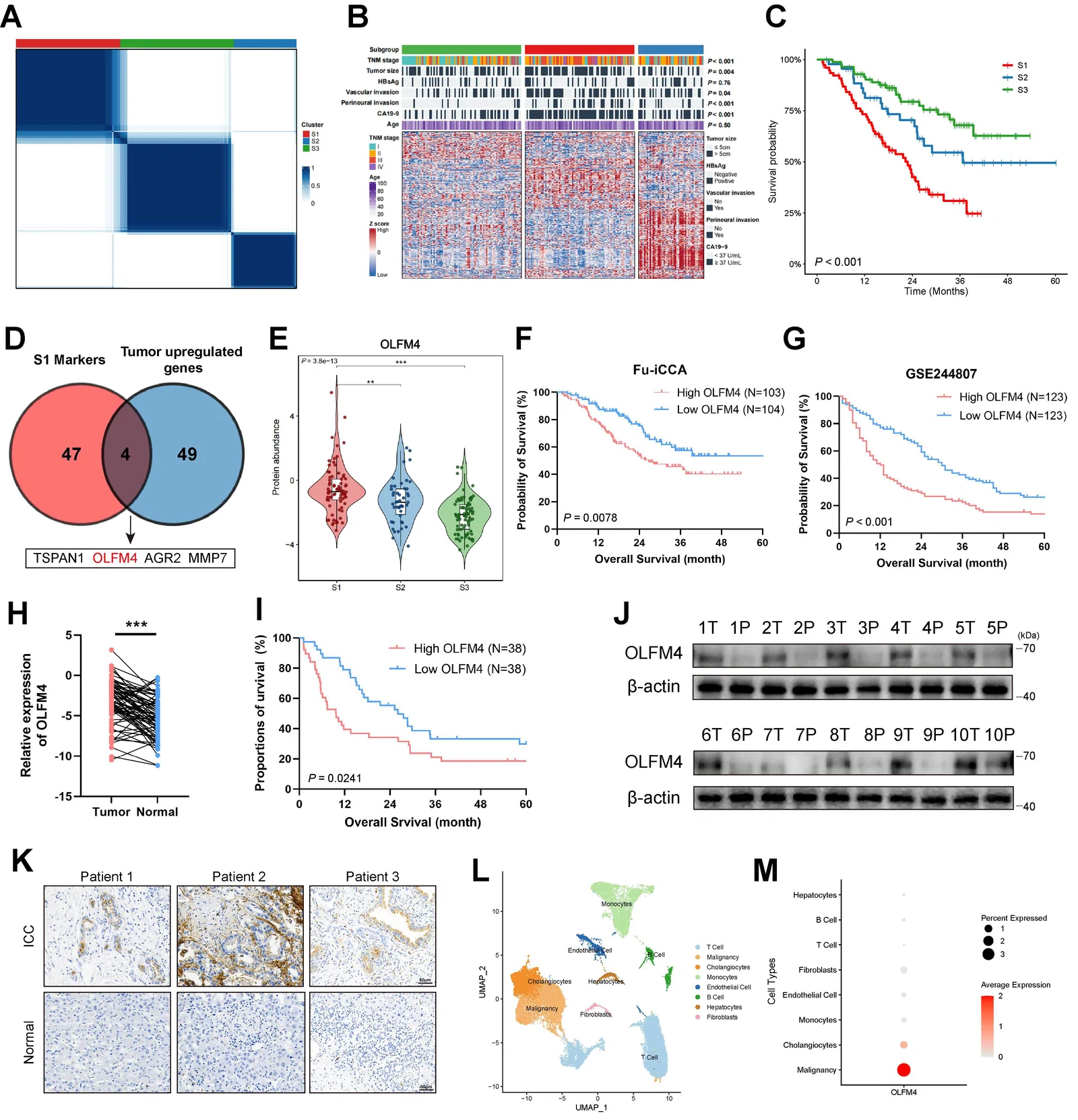
** OLFM4 is overexpressed in ICC and correlates with poor prognosis. (A)** Consensus clustering analysis classified the differential proteins into three metabolic subtypes (S1-S3). **(B)** Heatmap showing patient subgroups based on the most variable proteins. The significance of clinical features across proteomic subgroups was assessed using the Kruskal-Wallis rank-sum test for continuous variables and Fisher's exact test for categorical variables. **(C)** Kaplan-Meier overall survival analysis of patients stratified by metabolic subtype (S1-S3). **(D)** Venn diagram showing overlapping candidates between S1 marker proteins and upregulated genes in ICC from the GSE76297 dataset. **(E)** Relative abundance of OLFM4 across the subtypes. **(F, G)** Kaplan-Meier analysis of overall survival in patients with ICC based on OLFM4 expression levels. **(H)** OLFM4 mRNA levels in ICC and adjacent normal tissues were determined by qRT-PCR. **(I)** Kaplan-Meier analysis of overall survival based on OLFM4 levels (n = 76). **(J)** OLFM4 protein levels in ICC and adjacent normal tissues were determined by Western blotting. **(K)** Representative immunohistochemistry (IHC) staining of OLFM4 in paired ICC and adjacent normal tissues. Scale bar, 50 μm. **(L)** Uniform manifold approximation and projection (UMAP) showing the cell types in the ICC scRNA-seq dataset obtained from GSE138709. **(M)** Dot plot of OLFM4 expression in each cell type. *P < 0.05, **P < 0.01, ***P < 0.001.

**Figure 2:**
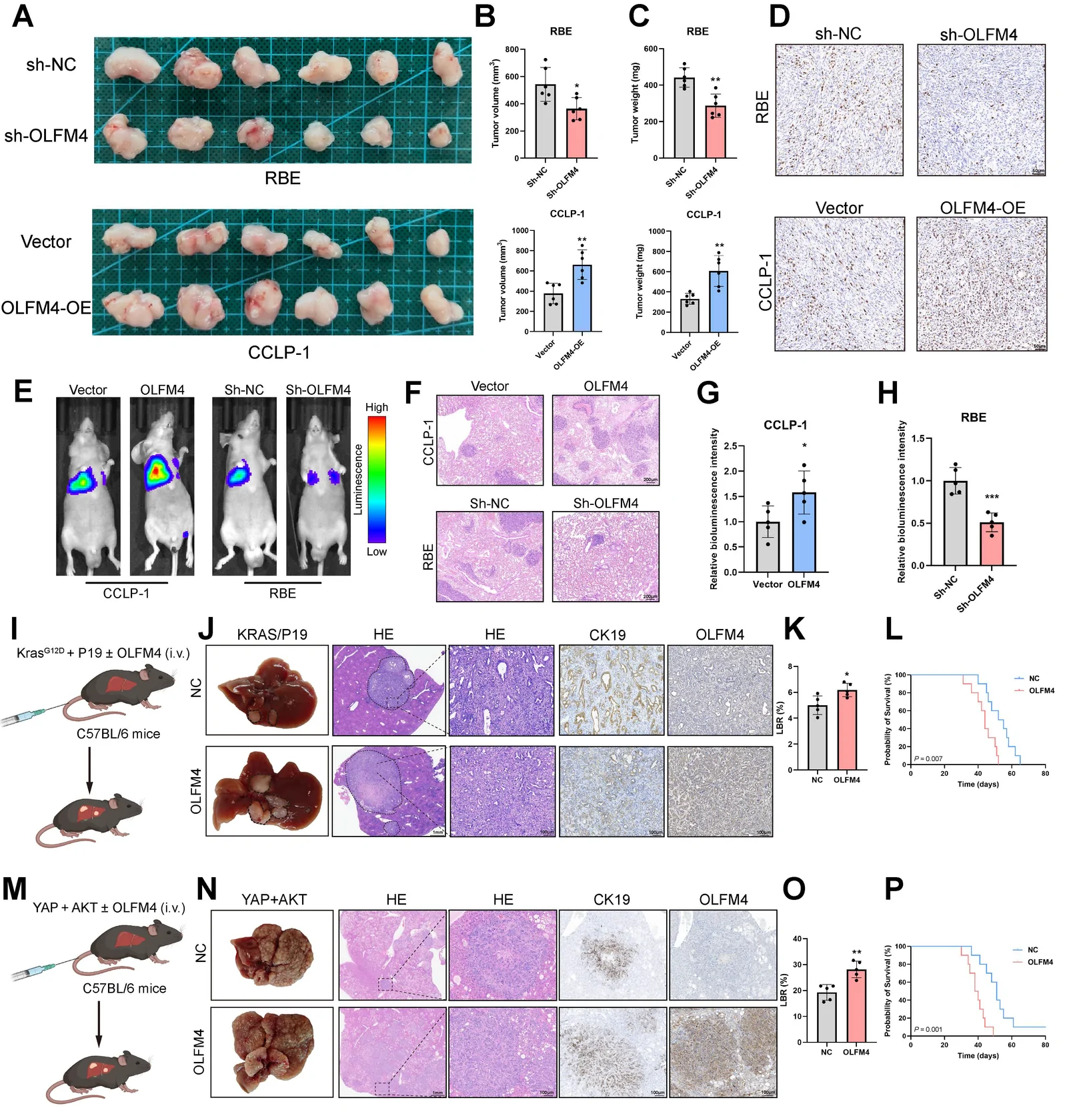
** OLFM4 promotes ICC progression *in vivo*. (A)** Subcutaneous tumors were established in mice with RBE cells with OLFM4 knockdown or CCLP-1 cells with OLFM4 overexpression. Representative images of subcutaneous xenograft tumors are shown (n = 6). **(B)** Volumes of subcutaneous xenograft tumors showing the effect of OLFM4 knockdown or overexpression on the formation of ICC. **(C)** Weight of subcutaneous xenograft tumors showing the effect of OLFM4 knockdown or overexpression on the formation of ICC.** (D)** Ki-67 staining of subcutaneous xenograft tumors. Scale bar, 50 μm. **(E)** Bioluminescence images of mice injected with transfected CCLP-1 or RBE cells via tail vein injection (n = 5). **(F)** Hematoxylin and eosin (H&E) staining of lung metastasis tumors. Scale bar, 200 μm. **(G, H)** Relative bioluminescence intensity for mice injected with CCLP-1 or RBE-transfected cells. **(I)** Schematic diagram of the ICC mouse model induced by KRAS/P19, with or without OLFM4 plasmid. Created with BioRender.com. **(J)** Representative images, H&E images and IHC images of CK19 and OLFM4 in the KRAS/P19 models (n = 5). Scale bars, 1 mm and 100 μm for whole and magnified images, respectively. **(K)** The liver/body weight ratio in the KRAS/P19 model mice. **(L)** Survival curve of the KRAS/P19 models (n = 10). **(M)** Schematic diagram of the ICC mouse model induced by YAP/AKT, with or without OLFM4 plasmid. Created with BioRender.com. **(N)** Representative images, H&E images and IHC images of CK19 and OLFM4 in the YAP/AKT models (n = 5). Scale bars, 1 mm and 100 μm for whole and magnified images, respectively. **(O)** The liver/body weight ratio in the YAP/AKT model mice. **(P)** Survival curve of the YAP/AKT models (n = 10). *P < 0.05, **P < 0.01, ***P < 0.001.

**Figure 3:**
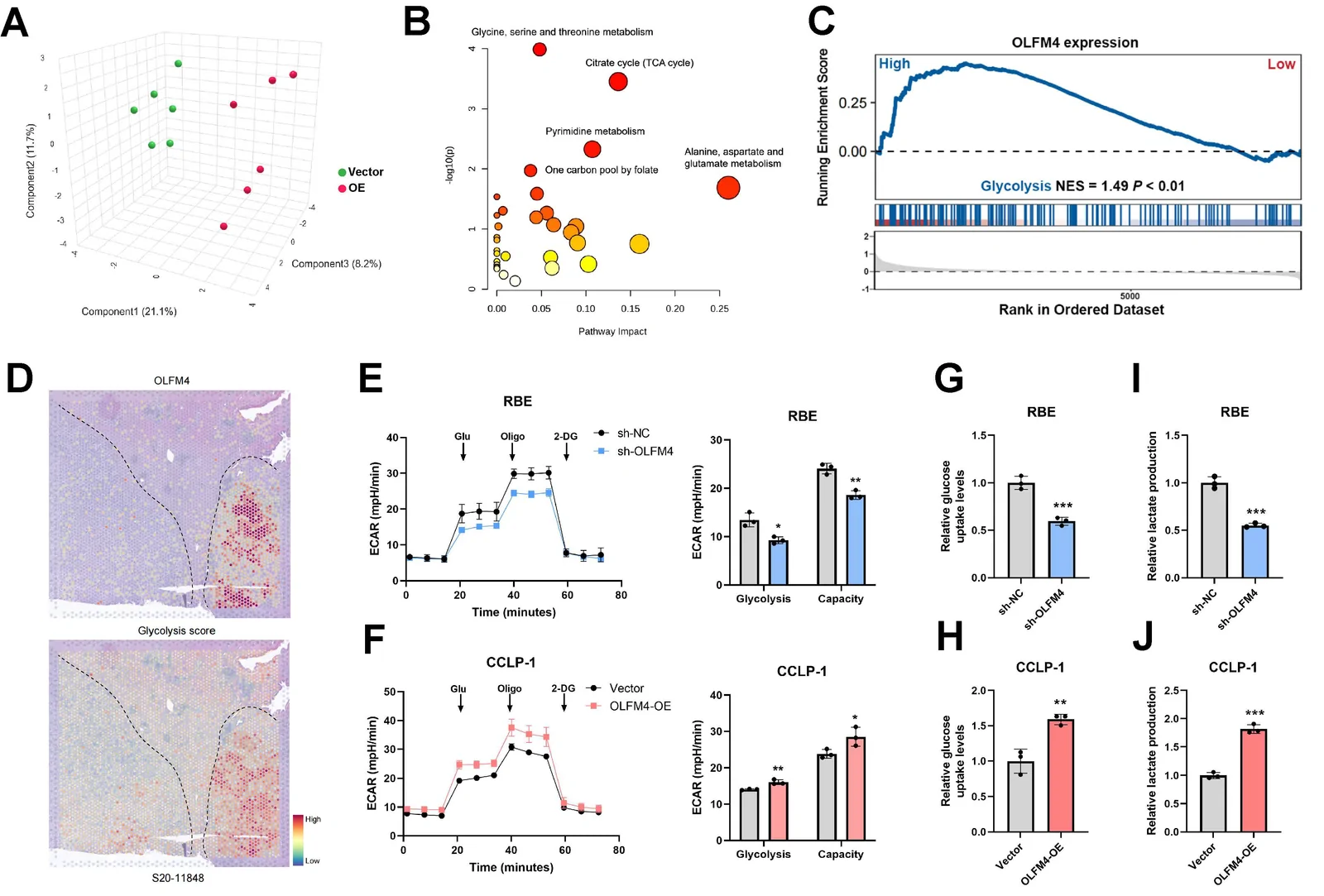
** OLFM4 promotes glycolysis in ICC. (A)** PLS-DA of metabolomics. **(B)** Pathway enrichment analysis of significantly altered metabolites. **(C)** Gene set enrichment analysis (GSEA) showing the relationship between OLFM4 expression and the glycolysis pathway. **(D)** Spatial plots of OLFM4 expression and glycolysis score in ICC.** (E, F)** Extracellular acidification rate (ECAR) levels of RBE or CCLP-1 cells were measured (left). Glycolysis rate and glycolytic capacity were analyzed (right). **(G, H)** Relative glucose uptake levels of RBE or CCLP-1 cells were measured. **(I, J)** Relative lactate levels of RBE or CCLP-1 cells were measured. *P < 0.05, **P < 0.01, ***P < 0.001.

**Figure 4:**
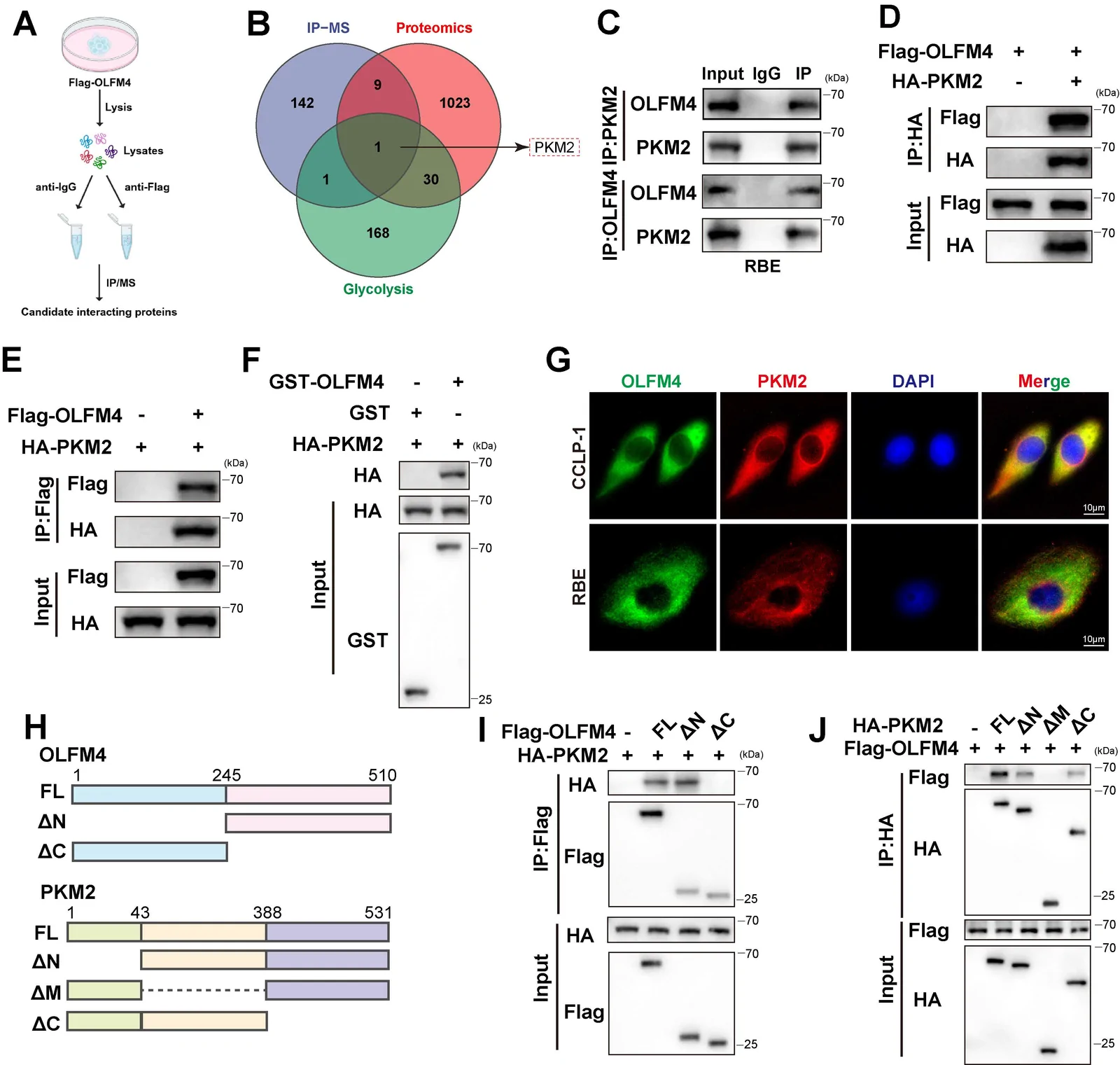
** OLFM4 interacts with PKM2 to regulate glycolysis in ICC. (A**) Schematic diagram illustrating IP-MS analysis to identify the interacting proteins of OLFM4. Created with BioRender.com. **(B)** Overlap of results from proteomics, IP-MS and glycolysis-related proteins. **(C)** Co-IP assays were performed to examine the interaction between OLFM4 and PKM2 in RBE cells. **(D, E)** Co-IP assays were performed to examine the interaction between OLFM4 and PKM2 in HEK293T cells. **(F)** GST pull-down assays were conducted to investigate direct binding between OLFM4 and PKM2. **(G)** IF staining showing the co-localization of OLFM4 and PKM2 in ICC cells. Scale bar, 10 μm. **(H)** Schematic diagram illustrating the structural domains and truncated mutants of OLFM4 (top) and PKM2 (bottom). **(I, J)** Co-IP assays were conducted to investigate the binding regions of OLFM4 and PKM2.

**Figure 5:**
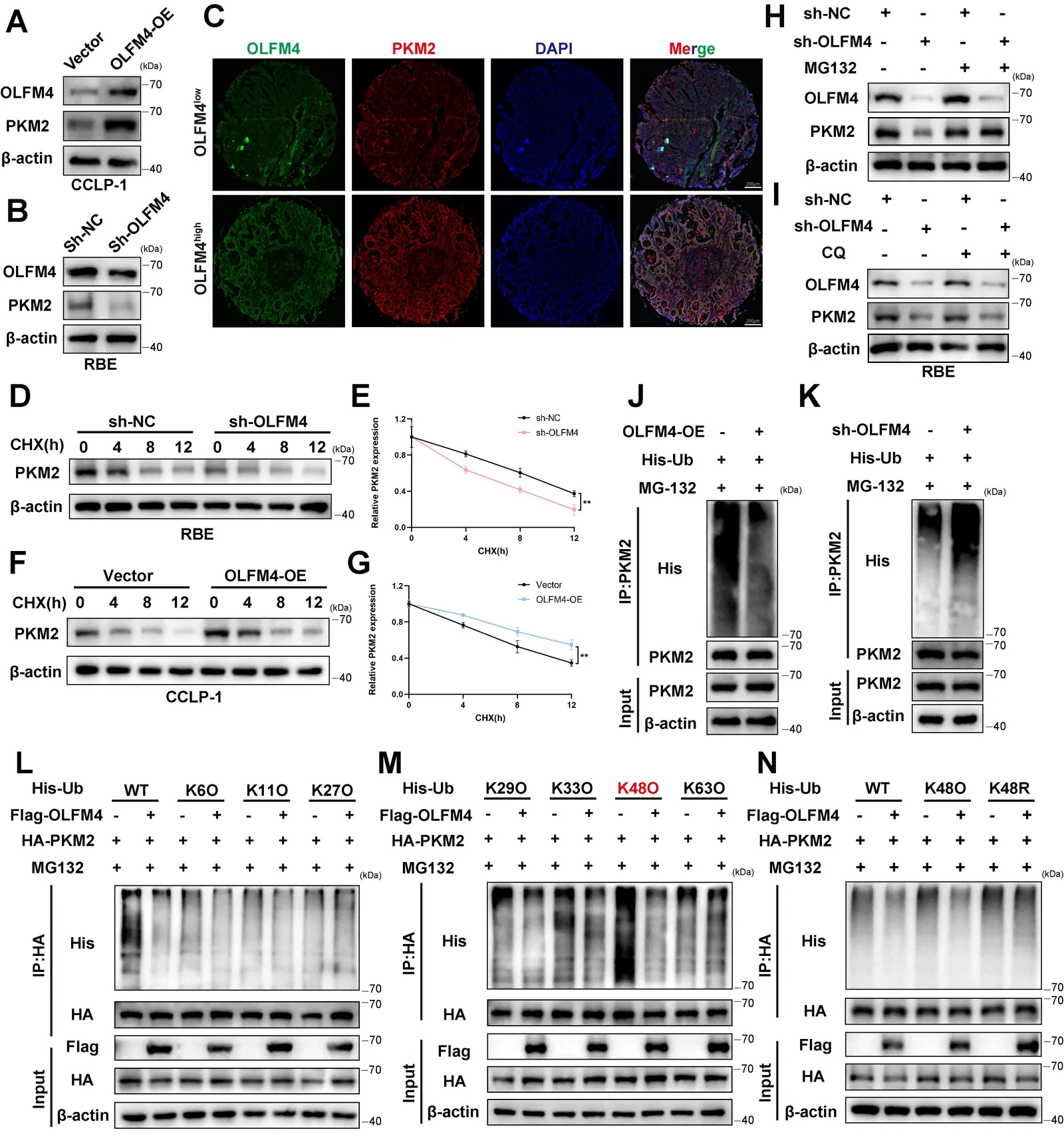
** OLFM4 inhibits K48-linked ubiquitin-mediated degradation of PKM2. (A, B)** The protein expression of PKM2 after OLFM4 overexpression or knockdown in ICC cells. **(C)** Representative mIHC images of OLFM4 and PKM2 staining in ICC tissues. Scale bar, 200 μm. **(D-G)** Expression levels of PKM2 in ICC cells treated with CHX for the indicated times. **(H, I)** The protein expression levels of PKM2 in ICC cells with OLFM4 knockdown were assayed following treatment with CQ or MG132. **(J, K)** The ubiquitination of PKM2 after OLFM4 knockdown or overexpression in ICC cells. **(L, M)** Screening of PKM2 ubiquitination regulated by OLFM4 using the indicated ubiquitin mutants (K6O, K11O, K27O, K29O, K33O, K48O, and K63O). **(N)** The ubiquitination of PKM2 in response to OLFM4 overexpression was investigated in HEK293T cells transfected with mutated His-Ub plasmids. *P < 0.05, **P < 0.01, ***P < 0.001.

**Figure 6:**
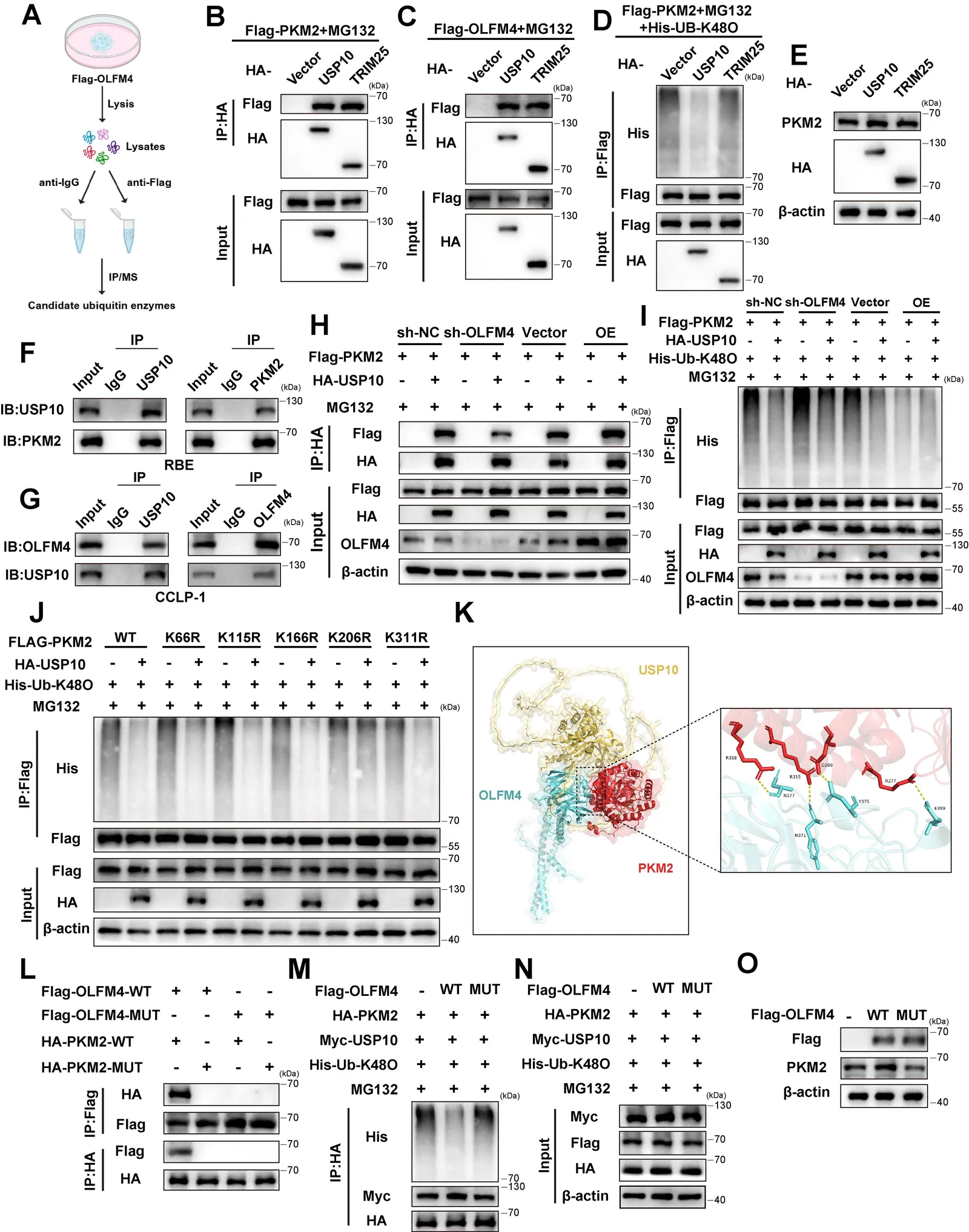
** OLFM4 enhances PKM2 deubiquitination by recruiting USP10. (A)** Schematic diagram illustrating the IP-MS analysis to identify candidate ubiquitin enzymes. Created with BioRender.com. **(B)** The interaction between PKM2 and USP10 or TRIM25 in HEK293T cells was analyzed using IP. **(C)** The interaction between OLFM4 and USP10 or TRIM25 in HEK293T cells was analyzed using IP. **(D)** K48-linked ubiquitination of PKM2 in HEK293T cells transfected with USP10 or TRIM25. **(E)** The protein expression levels of PKM2 in HEK293T cells transfected with USP10 or TRIM25. **(F, G)** Co-IP assays were conducted to investigate the interaction between USP10 and OLFM4 or USP10 and PKM2 in ICC cells. **(H)** The interaction of PKM2 and USP10 in OLFM4 knockdown and overexpression cells. **(I)** K48-linked ubiquitination of PKM2 in OLFM4 knockdown and overexpression cells. **(J)** Ubiquitination levels of PKM2 mutants in HEK293T cells transfected with the mutated Flag-PKM2 plasmids. **(K)** The simulated 3D structure of the OLFM4/PKM2/USP10 complex. Docking analysis revealed specific binding sites (BS) between OLFM4 and PKM2. **(L)** IP analysis showing the interaction of Flag-OLFM4-BS-WT or Flag-OLFM4-BS-MUT with HA-PKM2-BS-WT or HA-PKM2-BS-MUT in HEK293T cells. **(M, N)** Ubiquitination of PKM2 in HEK293T cells transfected with Flag-OLFM4-BS-WT or Flag-OLFM4-BS-MUT. **(O)** PKM2 expression levels in HEK293T cells transfected with Flag-OLFM4-BS-WT or Flag-OLFM4-BS-MUT.

**Figure 7:**
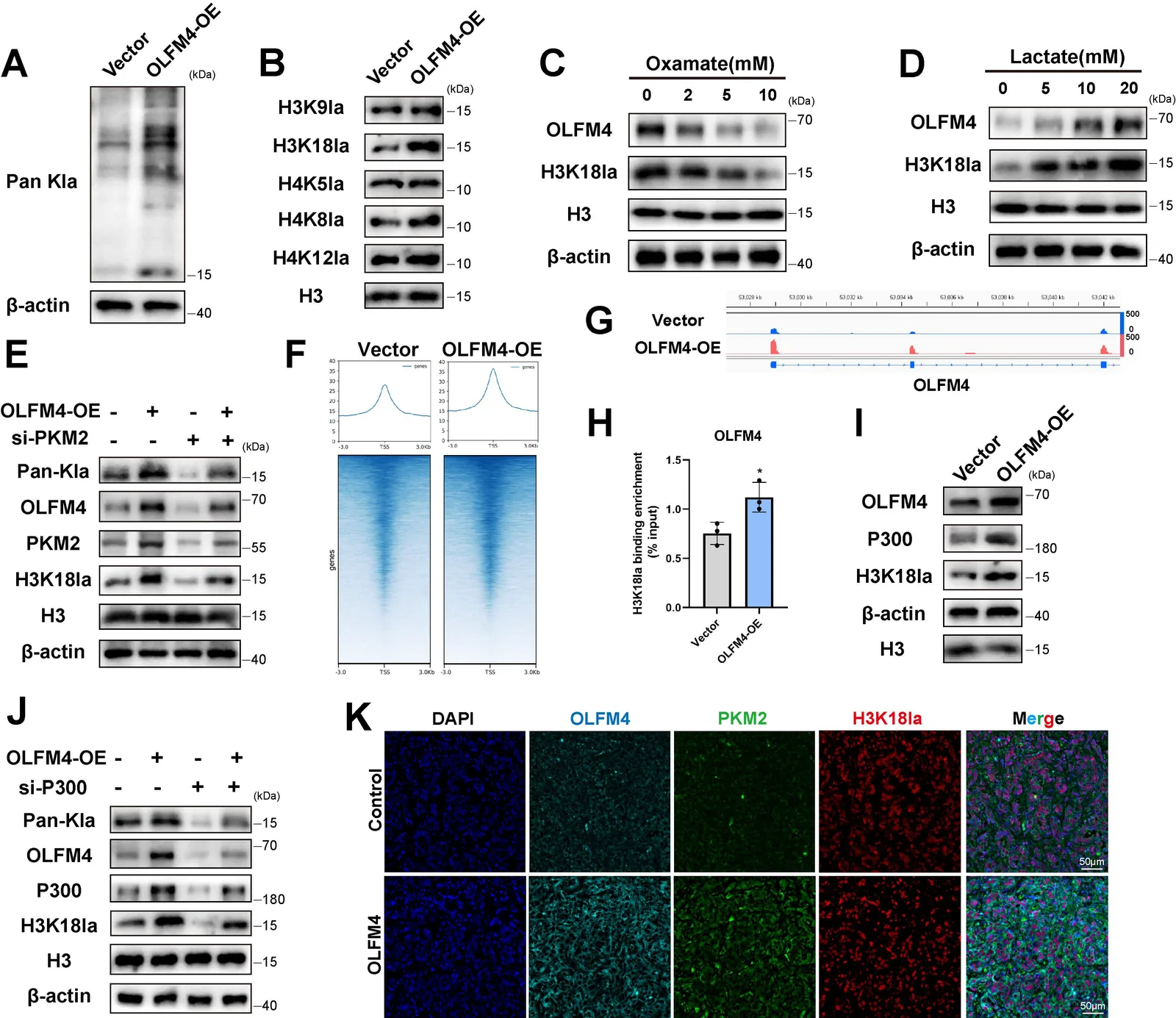
** OLFM4 enhances histone lactylation and H3K18la positively regulates OLFM4 expression. (A)** Pan-Kla levels in ICC cells transfected with vector or OLFM4 overexpression. **(B)** H3K9la, H3K18la, H4K5la, H4K8la, and H4K12la levels were measured by Western blotting. **(C, D)** Western blotting analysis of OLFM4 and H3K18la levels measured from ICC cells cultured in different concentrations of oxamate **(C)** and lactate **(D)** for 24 hours.** (E)** PKM2 knockdown attenuated the increase in Pan-Kla and H3K18la induced by OLFM4 overexpression in ICC cells. **(F)** The binding density of H3K18la was visualized using deepTools, showing the CUT&Tag tag counts at different H3K18la binding peaks in ICC cell lines treated with or without OLFM4 overexpression. **(G)** IGV snapshots showing H3K18la enrichment around the transcription start site (TSS) of OLFM4. **(H)** ChIP-qPCR analysis indicated the enrichment of H3K18la on the promoter regions of OLFM4 in ICC cells. **(I)** Western blotting analysis showing the levels of P300 in OLFM4 overexpression ICC cells. **(J)** Western blotting analysis showing the levels of Pan-Kla and H3K18la in ICC cells co-transfected with OLFM4 overexpression or control together with P300 knockdown or control. **(K)** Representative mIHC staining of OLFM4, PKM2 and H3K18la in mouse ICC tissues. Scale bar, 50 μm. *P < 0.05, **P < 0.01, ***P < 0.001.

**Figure 8:**
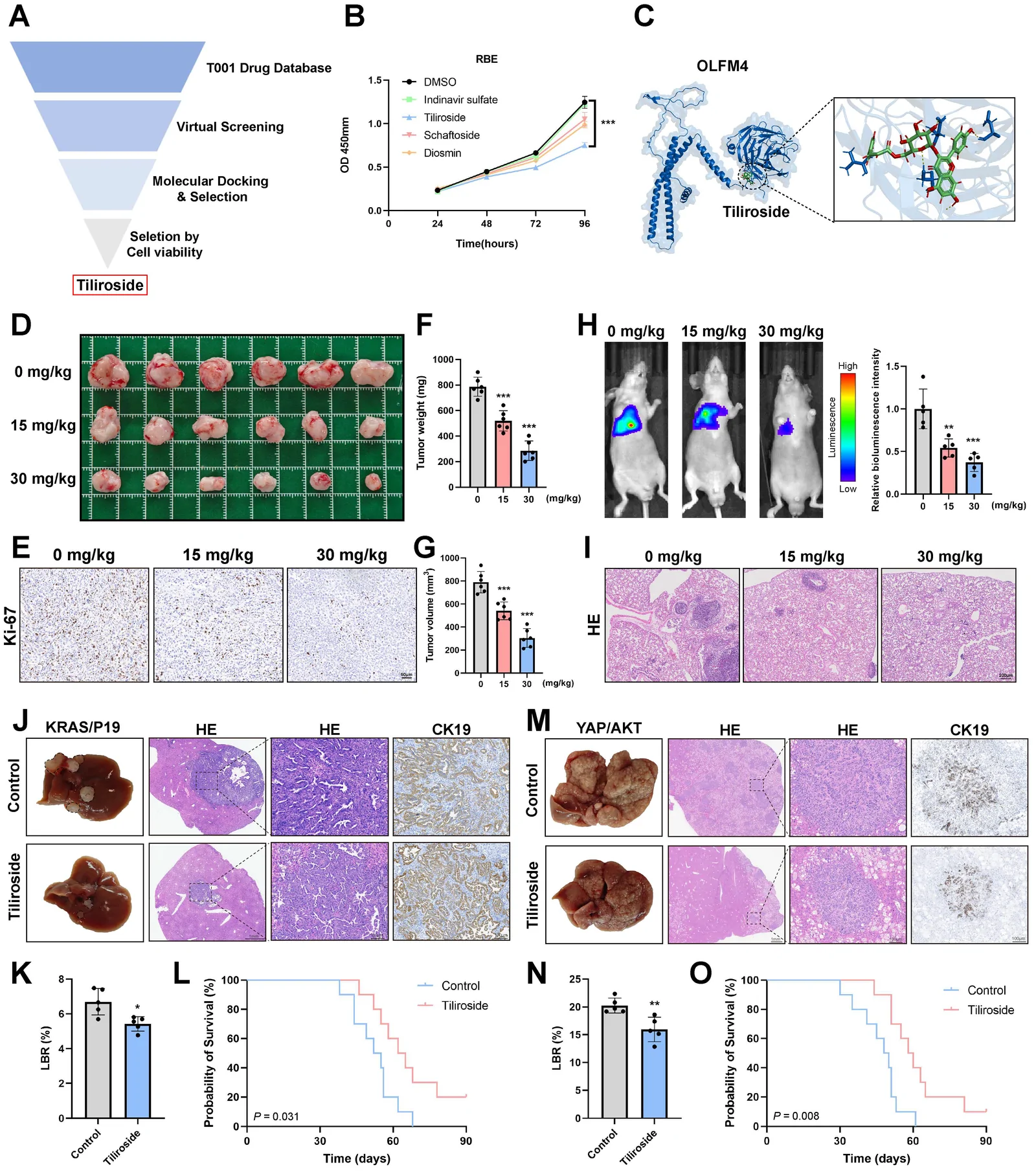
** Tiliroside is identified as a potential OLFM4 inhibitor in ICC. (A)** Schematic diagram of the strategy used to screen potential OLFM4 inhibitors in T001 library. **(B)** Cell viability of ICC cells treated with the four candidate drugs (20 µM) respectively, as determined by CCK-8 assay. **(C)** Docking analysis showing the interaction between tiliroside and OLFM4.** (D)** Subcutaneous tumors were established in mice treated with tiliroside at 0, 15, or 30 mg/kg (n = 6). **(E)** Ki-67 staining of subcutaneous xenograft tumors. Scale bar, 50 μm. **(F)** Tumor weights of the indicated subcutaneous xenografts.** (G)** Tumor volumes of the indicated subcutaneous xenografts. **(H)** Representative bioluminescence image of mice treated with tiliroside at 0, 15, or 30 mg/kg (left, n = 5). Quantification of relative bioluminescence intensity of lung metastatic tumors in mice (right). **(I)** Hematoxylin and eosin (H&E) staining of lung metastasis tumors. Scale bar, 200 μm. **(J)** Representative images, H&E images and IHC images of CK19 in the KRAS/P19 models with or without tiliroside treatment. Scale bars, 1 mm and 100 μm for whole and magnified images, respectively. **(K)** The liver/body weight ratio in the KRAS/P19 model mice. **(L)** Survival curve of the KRAS/P19 models (n = 10). **(M)** Representative images, H&E images and IHC images of CK19 in the YAP/AKT models with or without tiliroside treatment. Scale bars, 1 mm and 100 μm for whole and magnified images, respectively. **(N)** The liver/body weight ratio in the YAP/AKT model mice (n = 5). **(O)** Survival curve of the YAP/AKT models (n = 10). *P < 0.05, **P < 0.01, ***P < 0.001.

## Data Availability

The FU-iCCA proteomic is accessible through the biosino NODE database (OEP001105). Public bulk transcriptomic datasets were obtained from The Cancer Genome Atlas (TCGA) and Gene Expression Omnibus (GEO) database (GSE76297 and GSE244807). The public ICC single-cell dataset was downloaded from the GEO database (GSE138709). Spatial transcriptomic data were sourced from the Genome Sequence Archive (GSA; HRA000437) and from the HTAN dbGaP study accession phs002371.v3.p1. Further data supporting the findings of this study can be provided by the corresponding author upon reasonable request.

## Abbreviations

2-DG: 2-deoxy-D-glucose
CCK-8: Cell Counting Kit-8
ChIP-qPCR: chromatin immunoprecipitation-quantitative PCR
CHX: cycloheximide
CK19: cytokeratin 19
Co-IP: co-immunoprecipitation
CQ: chloroquine
CUT&Tag: cleavage under targets and tagmentation
ECAR: extracellular acidification rate
GSEA: gene set enrichment analysis
GST: glutathione S-transferase
GSVA: gene set variation analysis
H3K18la: histone H3 lysine 18 lactylation
Human-GEM: human genome-scale metabolic model
ICC: intrahepatic cholangiocarcinoma
IF: immunofluorescence
IGV: Integrative Genomics Viewer
IHC: immunohistochemistry
IP-MS: immunoprecipitation coupled with mass spectrometry
KEGG: Kyoto Encyclopedia of Genes and Genomes
LBR: liver/body weight ratio
mIHC: multiplex immunohistochemistry
NMF: non-negative matrix factorization
OLFM4: olfactomedin 4
OS: overall survival
P300: E1A binding protein p300
Pan-Kla: pan-lysine lactylation
PKM2: pyruvate kinase M2
SB: Sleeping Beauty
TCGA: The Cancer Genome Atlas
USP10: ubiquitin specific peptidase 10
