# Supplementary material for: OLFM4 promotes intrahepatic cholangiocarcinoma progression through a PKM2-glycolysis-histone lactylation feedback loop
Source: Int J Biol Sci. 2026 Sep 18;22(15):8333–47. doi: 10.7150/ijbs.135505 (PMC13685613; doi:10.7150/ijbs.135505)
Supplement: Supplementary file 1 [file ijbsv22p8333s1.pdf]

Materials and Methods

Clinical ICC specimens

Specimens were obtained from 76 ICC patients who underwent surgery at the First Affiliated Hospital with Nanjing Medical University. The use of clinical samples was approved by the Ethics Committee of hospital, and written informed consent was acquired from all participants in accordance with regional regulations.

Cell culture and transfection

HiBEC, RBE, HCCC-9810, HuCCT1 and HEK293T cells were purchased from the Cell Bank of the Chinese Academy of Sciences (Shanghai, China). CCLP-1 cells were kindly provided by Dr. Jijun Shan (Fudan University, Shanghai, China). Cells were cultured in DMEM (Gibco, Grand Island, NY, USA) containing 10% fetal bovine serum (FBS) and 1% penicillin-streptomycin at 37°C in a humidified incubator with 5% CO_2 (Thermo Fisher Scientific, Waltham, MA, USA).

Lentiviral vectors encoding target genes, shRNAs, and corresponding empty control vectors were designed and synthesized by Genomeditech (Shanghai, China). Stable cell lines were selected using 5 μg/mL puromycin (Beyotime, Shanghai, China) for 7 days. Plasmids and siRNAs were synthesized by GenePharma (Shanghai, China). Lipofectamine® 3000 reagent (Invitrogen, Carlsbad, CA, USA) was employed to deliver the indicated plasmids or siRNAs according to the manufacturer's protocol. Knockdown and overexpression efficiencies were verified by qRT–PCR and Western blotting. The sequences of the siRNAs and shRNAs in this study are listed in Table S3.

Animal experiments

Wild-type C57BL/6 mice and nude mice were obtained from the Animal Center of Nanjing Medical University. All animal studies were approved by the Nanjing Medical University Ethics Committee (No. 2409092).

For the subcutaneous xenograft assay, 100 μl of cell suspensions containing 5×10^6 stable CCLP-1 or RBE cells were subcutaneously injected into the axilla of

4-week-old male nude mice. Four weeks after injection, the mice were sacrificed. Tumor volumes were calculated using the formula: ($width^2$ × length) / 2, and tumors were weighed.

For the lung metastasis assay, 5×10^5 luciferase-labeled ICC cells in 100μL PBS were injected into the tail vein of nude mice. Four weeks later, D-luciferin (GoldBio, USA) was administered intraperitoneally, and bioluminescence signals were detected using an IVIS 100 Imaging System (Xenogen, Hopkinton, MA, USA).

For tiliroside treatment, mice were randomly divided into three groups: vehicle, 15 mg/kg tiliroside, and 30 mg/kg tiliroside. Tiliroside was administered intraperitoneally once daily for 3 weeks. At the endpoint, blood and major organs were collected for serum biochemical analyses and histopathological examination.

To establish primary mouse ICC models, a hydrodynamic tail vein injection approach based on the Sleeping Beauty (SB) transposon system was used. Plasmids were prepared in 2 mL sterile 0.9% saline for each mouse. Each injection solution contained a combination of 20 μg pT3-EF1α-HA-myr-AKT and 20 μg pT3-EF1α-YAP^{S127A}, or 20 μg $KRAS^{G12D}$ and 20 μg sgP19 plasmids, along with 5 μg pCMV-SB13, with or without 20 μg pT3-EF1α-OLFM4 plasmid. Plasmids were injected into 5-week-old male C57BL/6 mice by tail-vein within 6 to 8 seconds.

Western blotting

Proteins were extracted from tissues or cells using RIPA Lysis Buffer supplemented with 1% PMSF and Protease Inhibitor Cocktail (Beyotime, Shanghai, China). Western blotting was performed according to standard procedures. Protein bands were visualized using an ultra-sensitive ECL chemiluminescent substrate (Biosharp, Hefei, China). The antibodies are listed in Table S2.

qRT-PCR

The extraction of total RNA from tissue and cells was performed using the RNA-Quick Purification Kit (Yishan, Shanghai, China) following the manufacturer's protocol. Complementary DNA (cDNA) was synthesized from total RNA utilizing the

HiScript III 1st Strand cDNA Synthesis Kit (Vazyme, Nanjing, China). Quantitative real-time PCR was carried out using AceQ qPCR SYBR Green Master Mix (Vazyme, Nanjing, China) on a 7900HT Fast Real-Time PCR system (Applied Biosystems, Foster City, CA, USA). The relative expression was normalized to β-actin and calculated using the $2^{-\Delta\Delta Ct}$ method. Primer information is listed in Table S3.

Cell proliferation assays

For Cell Counting Kit-8 (CCK-8) assays, cells were seeded into 96-well plates at an initial density of 1×10^3 cells per well. At the indicated time points, 10 μl of CCK-8 reagent (Dojindo, Kumamoto, Japan) was added to each well daily. After 2 h incubation, the absorbance at 450 nm was detected by Multi-Mode Microplate Reader (Biotek, Winooski, VT, USA).

For 5-Ethynyl-2'-deoxyuridine (EdU) assays, EdU cell proliferation kit (Beyotime, Shanghai, China) was used according to the manufacturer's instructions. The images were captured under a fluorescence microscope (Leica, Wetzlar, Germany).

For colony formation assays, the transfected cells were seeded in 6-well plates (1×10^3 cells/well) and cultured for 10 days, followed by fixation and staining with Crystal Violet Staining Solution (Beyotime, Shanghai, China).

Transwell and wound healing assays

For the Transwell assay, 5×10^4 ICC cells suspended in serum-free DMEM were seeded into the upper chambers, with DMEM containing 20% FBS placed in the lower chambers (Corning, NY, USA). After 2 days, cells that had migrated through the membrane were fixed, stained with crystal violet, and photographed using a light microscope (Olympus, Tokyo, Japan).

For the wound healing assay, ICC cells were cultured in six-well plates until reaching approximately 90% confluence. The cells were scratched with a 200-μL pipette tip, followed by incubation in serum-free medium. Images were acquired at 0 and 48 h under a light microscope (Olympus, Japan).

Cleavage Under Targets and Tagmentation (CUT&Tag) and ChIP–qPCR

Hyperactive Universal CUT&Tag Assay Kit for Illumina Pro (Vazyme) was used for CUT&Tag following the manufacturer's protocol. Libraries were sequenced by Novogene (Beijing, China). ChIP was carried out using a ChIP Assay Kit (Millipore, Billerica, MA, USA), and the precipitated DNA was purified and analyzed by qPCR.

Immunohistochemistry (IHC)

Tissue sections were deparaffinized, and subsequently rehydrated through graded ethanol. Antigen retrieval was conducted with citrate antigen retrieval solution (Beyotime, Shanghai, China), followed by blocking with QuickBlock™ Blocking Buffer for Immunol Staining (Beyotime, Shanghai, China) for 1 h at room temperature. The sections were then incubated with specific primary antibodies at 4°C overnight. After washing, the sections were incubated with the secondary antibody. Stained sections were photographed by a light microscope (Nikon, Tokyo, Japan).

Extracellular acidification rate (ECAR)

A Seahorse XF96 Extracellular Flux Analyzer (Agilent Technologies, Santa Clara, CA, USA) was employed to measure ECAR, using the XF Glycolysis Stress Test Kit following the manufacturer's guidelines.

Glucose uptake and lactate production

Glucose levels in ICC cells were measured by glucose assay kit (Nanjing Jiancheng Bioengineering Institute, Nanjing, China) according to the manufacturer's instructions. Lactate levels in ICC cells were evaluated with lactate assay kit (Nanjing Jiancheng Bioengineering Institute, Nanjing, China) following the manufacturer's instructions.

Co-Immunoprecipitation (Co-IP)

Cells were lysed in NP-40 substitute lysis buffer (Beyotime, Shanghai, China). Cell lysates were incubated with the antibodies or control IgG at 4 ℃ overnight with gentle rotation, followed by incubation with Protein A/G magnetic beads (Beyotime,

Shanghai, China) for 2 h at room temperature. The immunoprecipitated protein complexes were collected and separated by SDS-PAGE, and then analyzed by Western blotting.

Immunoprecipitation coupled with mass spectrometry (IP/MS)

Immunoprecipitated protein complexes were analyzed by mass spectrometry. Liquid chromatography-tandem mass spectrometry (LC-MS/MS) analysis and initial bioinformatics protein identification were performed by Hangzhou Cosmos Wisdom Biotech Co., Ltd.

Glutathione S-Transferase (GST) pull-down assay

HEK293T cells were transfected with GST-tagged plasmids or GST control and lysed in buffer supplemented with a protease inhibitor cocktail. The lysates were incubated with 30 μL of Glutathione Sepharose beads (Beyotime) for 3 h at 4 °C to capture GST-tagged proteins. Subsequently, the bound proteins were purified and eluted according to the manufacturer's instructions. The protein-bead complexes were washed and boiled at 95 °C for 10 min. Finally, the samples were analyzed by western blotting.

Multiplexed immunohistochemistry (mIHC)

Tissue sections were deparaffinized, rehydrated using alcohol, and subjected to antigen retrieval using citrate antigen retrieval solution (Beyotime, Shanghai, China). The sections were treated with 0.3% hydrogen peroxide to block endogenous peroxidase activity. Multiplex staining was performed using a 3-color fluorescence kit (Aifang Biotechnology, Changsha, China). Following incubation with appropriate primary antibodies, the sections were incubated with HRP-conjugated secondary antibodies, and this procedure was repeated for sequential rounds of single-marker staining. Nuclei were counterstained with DAPI, and slides were mounted and visualized using the Thunder Imaging System (Leica, Wetzlar, Germany).

Molecular docking

The protein crystal structure of PKM2 was retrieved from the RCSB Protein Data Bank (PDB ID: 1ZJH), and the protein structures of OLFM4 and USP10 were predicted using AlphaFold 3. The HDOCK server [1] was utilized to perform the computational protein-protein docking simulations. The structural models were visualized using PyMOL.

Virtual screening of potential OLFM4 inhibitors

Potential binding pockets were predicted based on the structure of OLFM4. Protein preparation was performed using the Protein Preparation Wizard in Schrödinger. Virtual screening against the predicted binding pockets was subsequently performed using the T001 library (TopScience), containing 3,927 compounds. Candidate compounds were prioritized based on docking score and MM/GBSA binding free values. The top four commercially available compounds were purchased from Targetmol for experimental validation.

Spatial transcriptome analysis

Spatial transcriptome sequencing data were obtained from the studies reported by Wu et al. and Mo et al. [2, 3]. Spot-level expression matrices from the ST and Visium platforms were processed in R with the Seurat package. Data were normalized using the SCTransform algorithm. Glycolysis scores for individual spots were calculated with the AddModuleScore function based on a glycolysis-related gene set. Spatial distributions of gene expression were visualized using the SpatialFeaturePlot function.

Single-cell RNA-sequencing (scRNA-seq) analysis

The scRNA-seq dataset (GSE138709) was downloaded from the GEO database and analyzed with Seurat according to the workflow described in our previous study [4].

General proteomic and metabolic subtyping

The abundance matrix of Human-GEM proteins in Fu-iCCA was min–max

normalized across samples and used as the input for non-negative matrix factorization (NMF). The cophenetic correlation coefficient reached its maximum at k = 3; therefore, three clusters were selected as the optimal candidate metabolic subtypes.

Public databases

MSigDB (http://www.gsea-msigdb.org/gsea/msigdb/) was used to obtain glycolysis-related gene sets. MetaboAnalyst (https://www.metaboanalyst.ca/) was used for KEGG pathway enrichment analysis of significantly altered metabolite clusters. PhosphoSitePlus (https://www.phosphosite.org/) and GPS-Uber [5] were used to predict potential lysine ubiquitination sites on PKM2.

Statistical analysis

Statistical analyses were performed using SPSS 27.0 and GraphPad Prism 9.0 software. Data are shown as the mean ± SD from at least three independent experiments. For continuous variables, comparisons between two groups were conducted using t test. The chi-square test was applied to evaluate the correlation between OLFM4 expression and various clinicopathological features. Non-normally distributed continuous variables were compared via the Wilcoxon rank-sum test. Differences in prognosis were determined by log-rank analysis of Kaplan-Meier survival curves. Statistical significance was defined as follows: $*P < 0.05$, $**P < 0.01$, $***P < 0.001$.

References

1. Yan Y, Tao H, He J, Huang SY. The HDOCK server for integrated protein-protein docking. Nat Protoc. 2020;15(5):1829-1852.

2. Wu R, Guo W, Qiu X, et al. Comprehensive analysis of spatial architecture in primary liver cancer. Sci Adv. 2021;7(51):eabg3750.

3. Mo CK, Liu J, Chen S, et al. Tumour evolution and microenvironment interactions in 2D and 3D space. Nature. 2024;634(8036):1178-1186.

4. Liu S, Fan S, Wang Y, et al. ACSL4 serves as a novel prognostic biomarker

correlated with immune infiltration in Cholangiocarcinoma. BMC Cancer. 2023;23(1):444.

5. Wang C, Tan X, Tang D, et al. GPS-Uber: a hybrid-learning framework for prediction of general and E3-specific lysine ubiquitination sites. Brief Bioinform. 2022;23(2):bbab574.

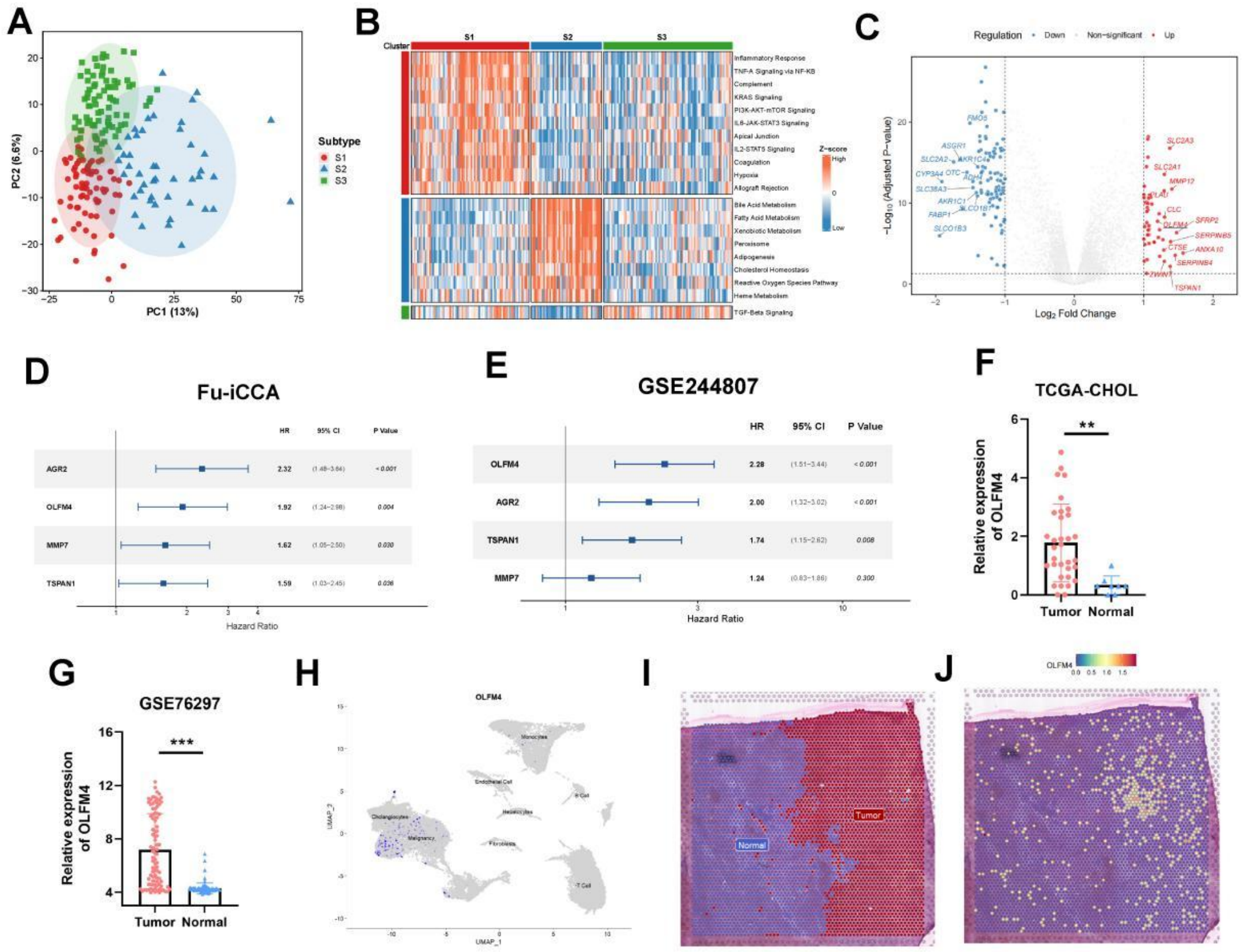

Figure S1 Identification of metabolic subtypes and candidate genes in ICC.

(A) Principal component analysis (PCA) plot showing distinct expression patterns among the subtypes. **(B)** GSVA showing pathway enrichment across the subtypes. **(C)** Volcano plot showing the S1 marker proteins. **(D, E)** Univariate Cox regression analysis of four candidate genes in the FU-iCCA cohort and the GSE244807 dataset. **(F)** Relative expression of OLFM4 in ICC and normal tissues in the TCGA-CHOL dataset. **(G)** Relative expression of OLFM4 in ICC and normal tissues in the GSE76297 dataset. **(H)** UMAP plots displaying the expression of OLFM4 across cell types. **(I)** Malignant and normal regions were identified in spatial transcriptomics. **(J)** Spatial feature plots showing the expression levels and distribution of OLFM4. *$P < 0.05$, **$P < 0.01$, ***$P < 0.001$.

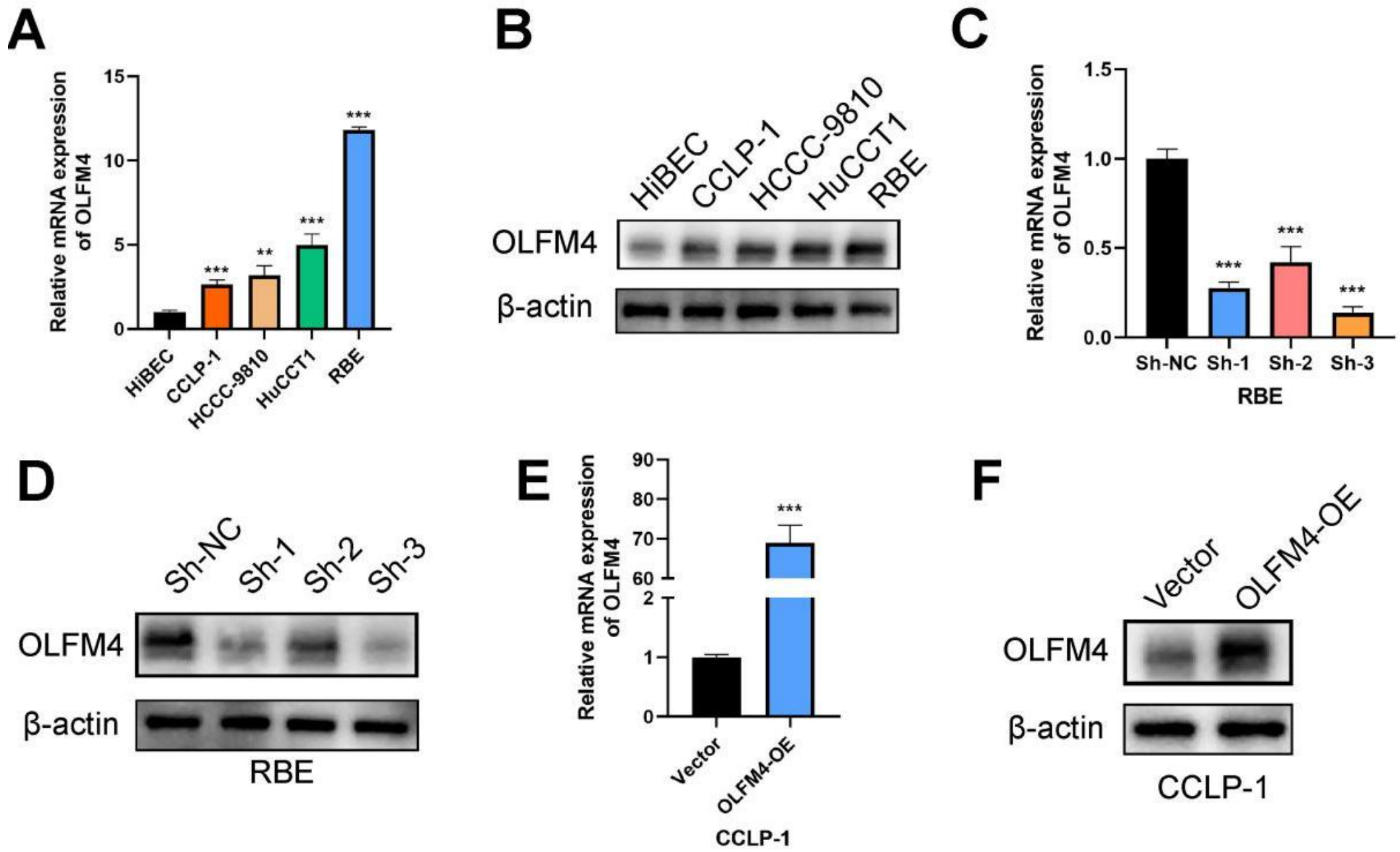

Figure S2 The Expression of OLFM4 in ICC cell lines

(A, B) OLFM4 mRNA and protein expression levels in HiBEC and ICC cell lines were determined by qRT–PCR and Western blotting analysis. **(C, D)** Validation of OLFM4 knockdown efficiency in RBE cells by qRT–PCR and Western blotting analysis. **(E, F)** Validation of OLFM4 overexpression efficiency in CCLP-1 cells by qRT–PCR and Western blotting analysis. *$P < 0.05$, **$P < 0.01$, ***$P < 0.001$.

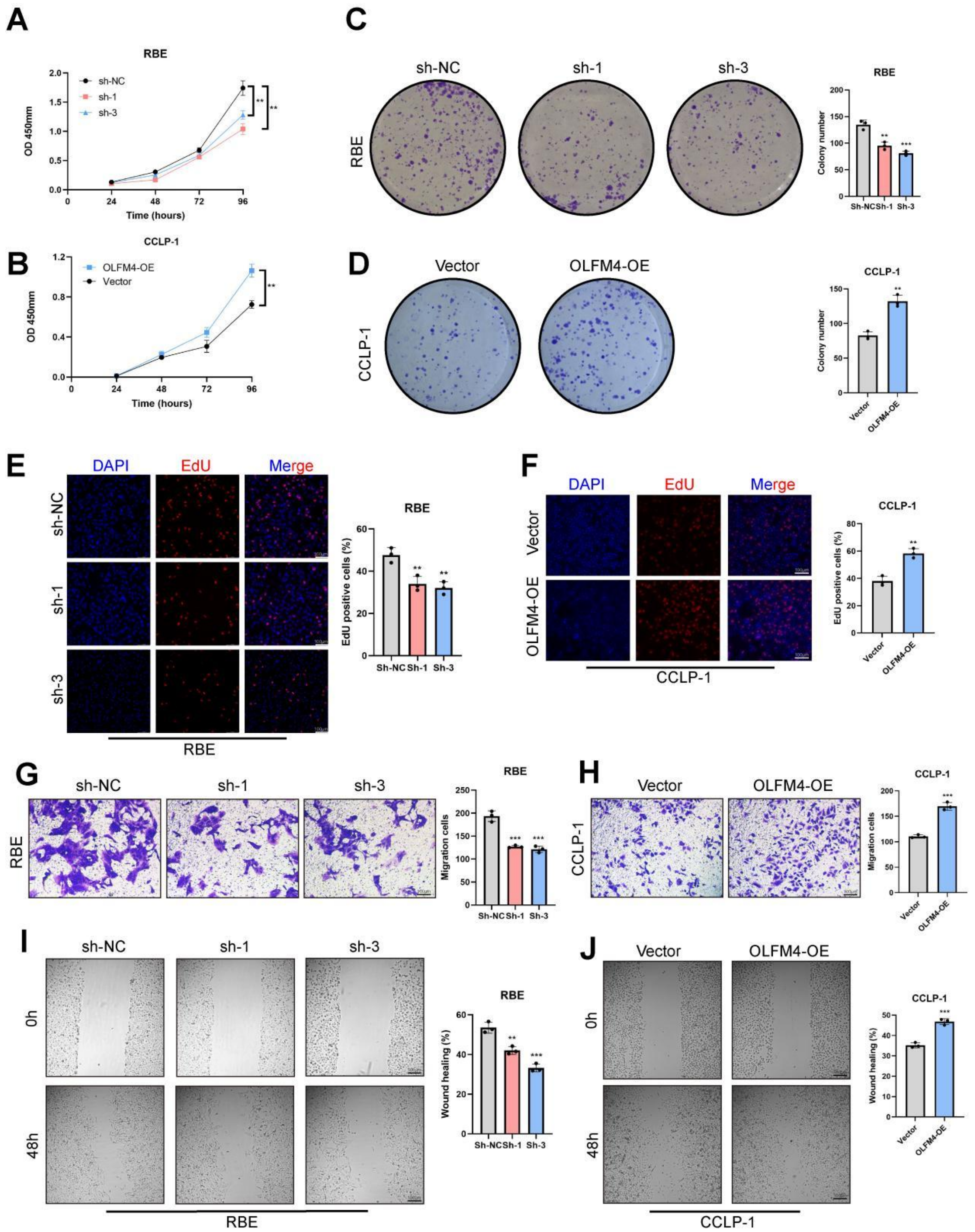

Figure S3 OLFM4 promotes the proliferation and migration of ICC cells *in vitro*. **(A, B)** CCK-8 assays assessing cell viability in RBE and CCLP-1 cells with OLFM4 knockdown or overexpression. **(C, D)** Colony formation assays assessing the proliferative capacity of RBE and CCLP-1 cells with OLFM4 knockdown or overexpression. **(E, F)** EdU assays evaluating proliferative capacity of RBE and CCLP-1 cells following OLFM4 knockdown or overexpression. **(G, H)** Transwell

assays measuring the migratory capacity of RBE and CCLP-1 cells with OLFM4 knockdown or overexpression. Scale bar, 100 μm. **(I, J)** Wound healing assays evaluating the migratory capacity of RBE and CCLP-1 cells with OLFM4 knockdown or overexpression. Scale bar, 100 μm. *$P < 0.05$, **$P < 0.01$, ***$P < 0.001$.

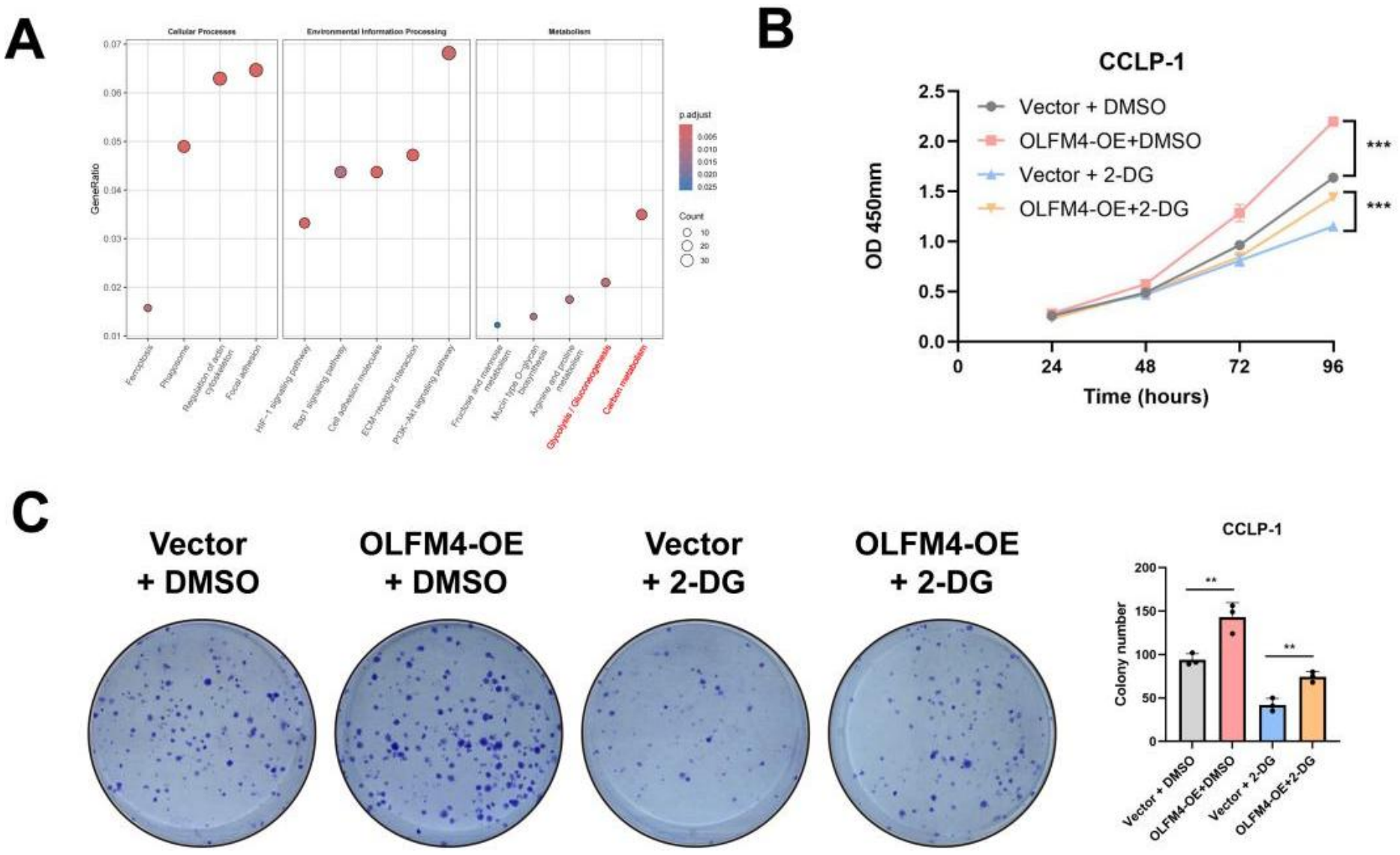

Figure S4 OLFM4 enhances glycolysis in ICC.

(A) KEGG pathway analysis of differentially expressed proteins between high and low OLFM4 expression group in the FU-iCCA proteomics cohort. **(B)** CCK-8 assays assessing cell viability in CCLP-1 cells with vector or OLFM4 overexpression, with or without 2-DG treatment. **(C)** Colony formation assays in CCLP-1 cells with OLFM4 overexpression, with or without 2-DG treatment. *$P < 0.05$, **$P < 0.01$, ***$P < 0.001$.

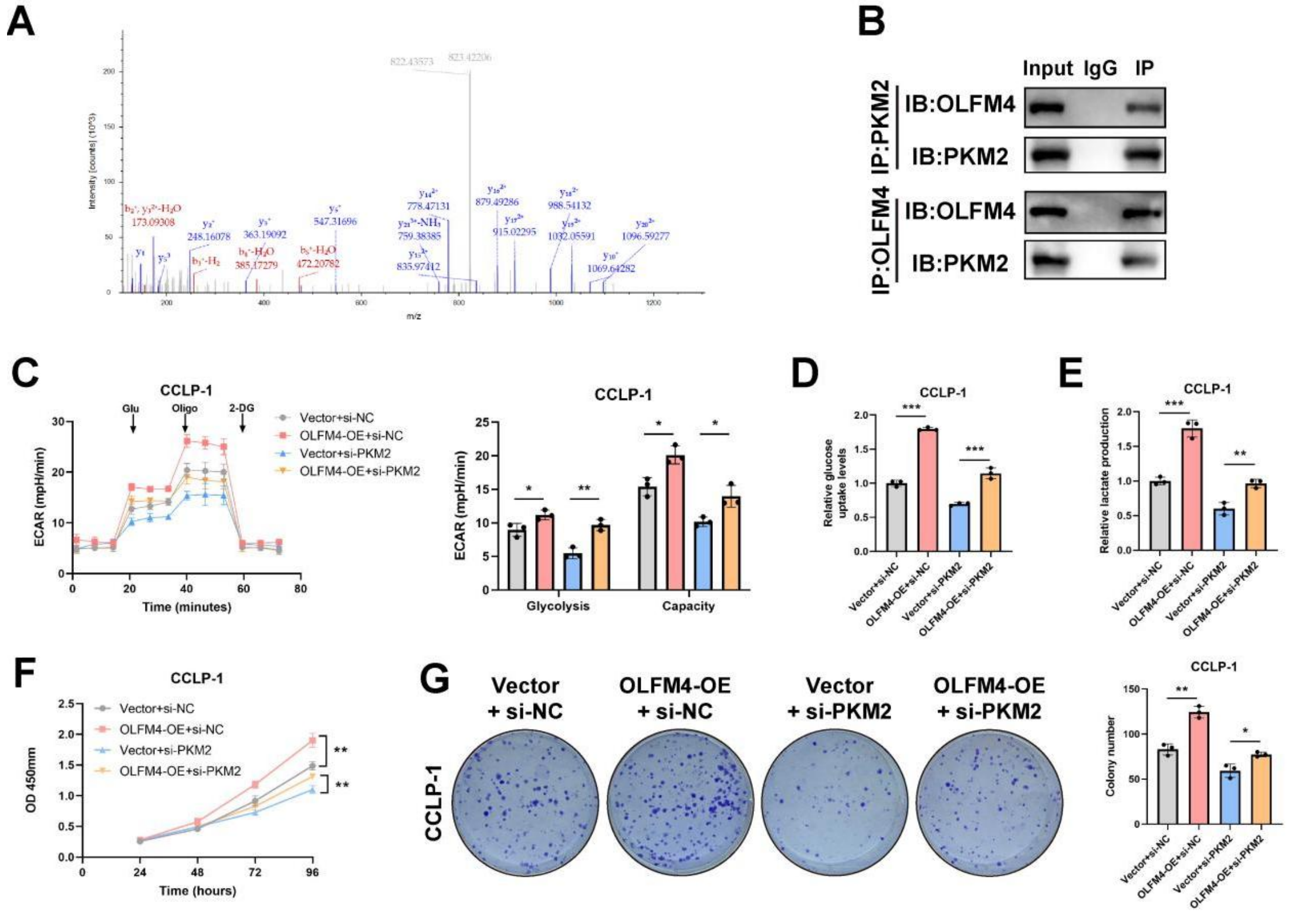

Figure S5 OLFM4 promotes glycolysis via interacting with PKM2 in ICC.

(A) Representative MS spectrum identifying the characteristic peptide of PKM2. **(B)** Co-IP assays were performed to examine the interaction between OLFM4 and PKM2 in CCLP-1 cells. **(C)** Extracellular acidification rate (ECAR) levels of CCLP-1 cells were measured (left). Glycolysis rate and glycolytic capacity were analyzed (right). **(D)** Relative glucose uptake levels of CCLP-1 cells were measured. **(E)** Relative lactate production of CCLP-1 cells were measured. **(F)** CCK-8 assays assessing cell viability in CCLP-1 cells. **(G)** Colony formation assays in CCLP-1 cells. *$P < 0.05$, **$P < 0.01$, ***$P < 0.001$.

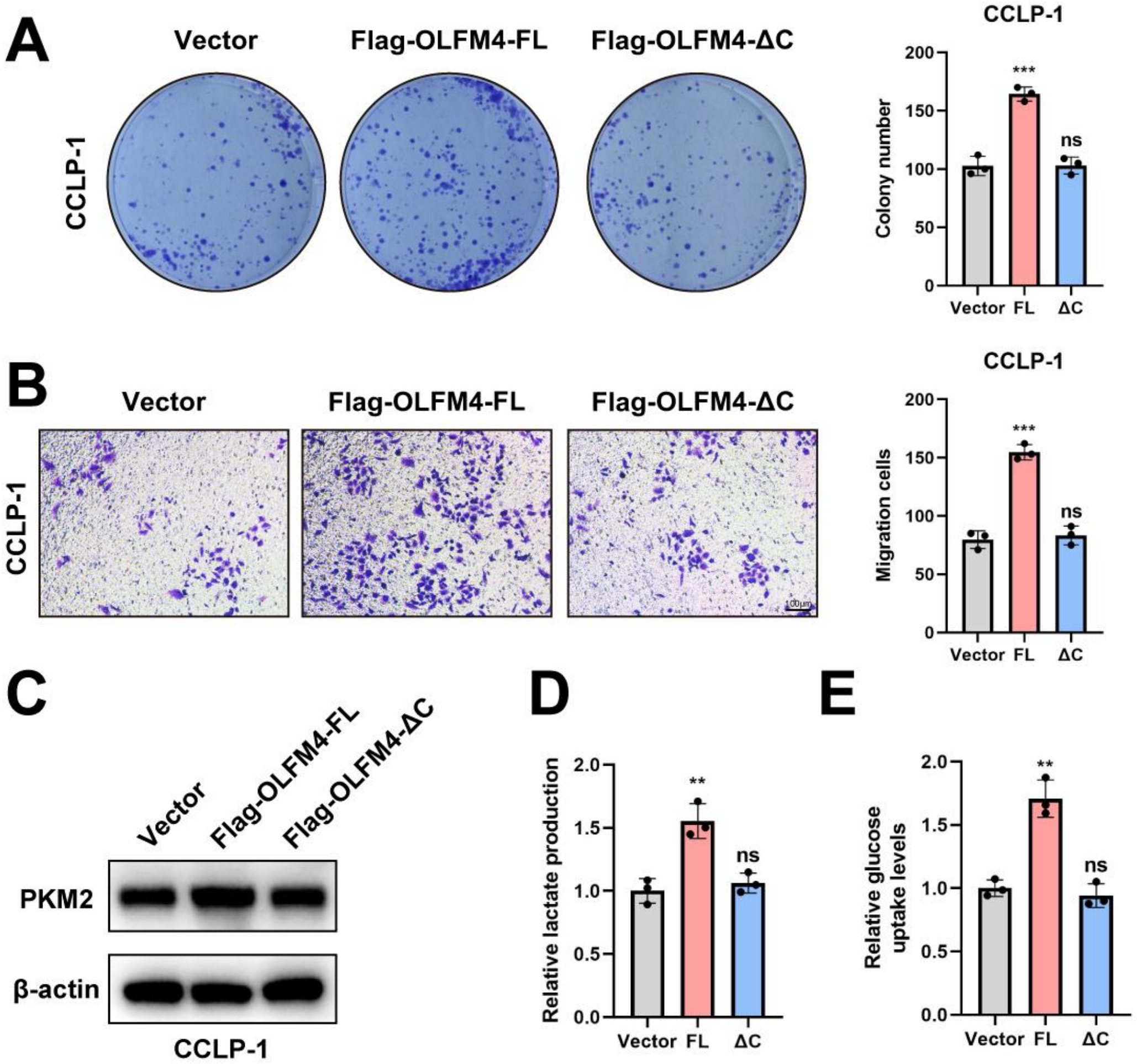

Figure S6 The C-terminal domain is essential for OLFM4 to promote ICC cell proliferation, migration, and glycolysis through its interaction with PKM2.

(A) Colony formation assays evaluating the clonogenic capacity of CCLP-1 cells transfected with Vector, Flag-OLFM4-FL, or Flag-OLFM4-ΔC. **(B)** Transwell migration assays in CCLP-1 cells. Scale bar, 100 μm. **(C)** Western blotting analysis of PKM2 protein levels in CCLP-1 cells transfected with Vector, Flag-OLFM4-FL, or Flag-OLFM4-ΔC. **(D, E)** Relative lactate production **(D)** and relative glucose uptake levels **(E)** in CCLP-1 cells under the indicated transfection conditions. *$P < 0.05$, **$P < 0.01$, ***$P < 0.001$, ns, not significant.

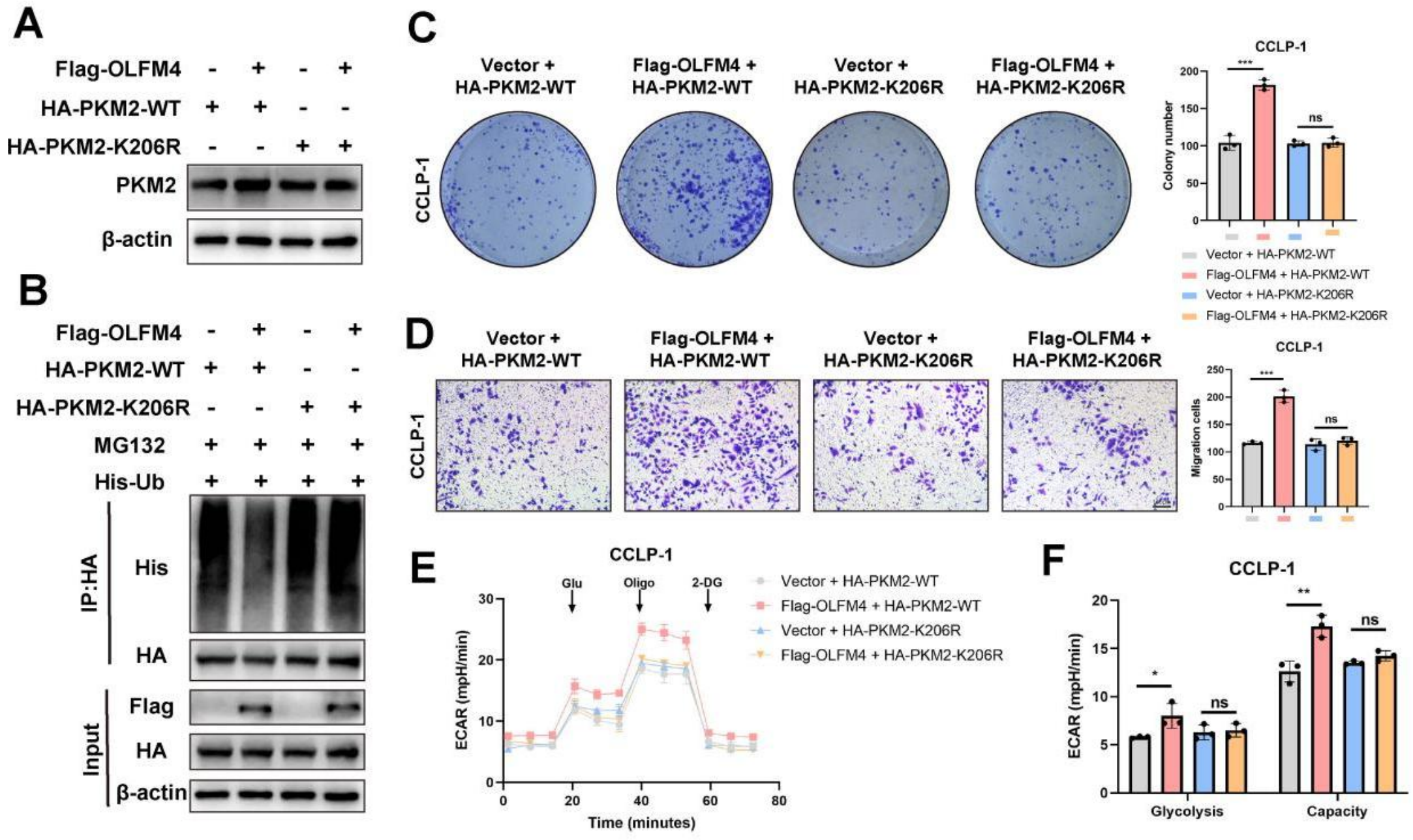

Figure S7 OLFM4 promotes ICC cell proliferation, migration, and glycolysis in a PKM2-K206-dependent manner.

(A) Western blotting analysis of PKM2 protein levels in CCLP-1 cells with stable PKM2 knockdown transfected with HA-PKM2-WT or HA-PKM2-K206R, with or without Flag-OLFM4 overexpression. **(B)** Ubiquitination levels of PKM2 in the indicated CCLP-1 cells. **(C)** Colony formation assays evaluating the proliferation of the indicated CCLP-1 cells. **(D)** Transwell assays evaluating migration in the indicated CCLP-1 cells. Scale bar, 100 μm. **(E, F)** ECAR **(E)** and quantification of glycolysis and glycolytic capacity **(F)** were analyzed. *P < 0.05, **P < 0.01, ***P < 0.001, ns, not significant.

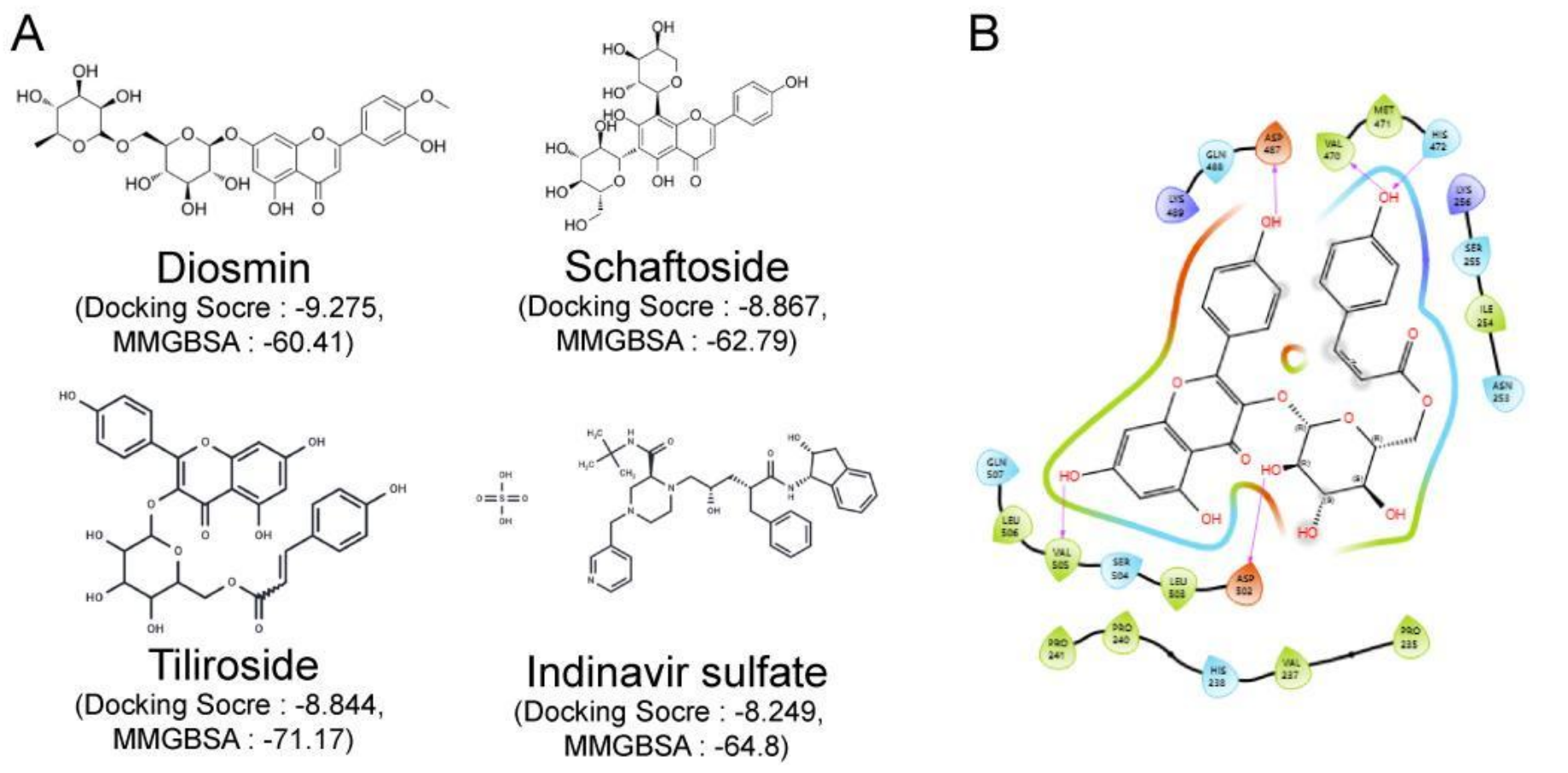

Figure S8 Tiliroside is identified as a potential OLFM4 inhibitor to suppress ICC malignant progression.

(A) The structures and docking score of four potential OLFM4 inhibitors identified from the T001 compound library. **(B)** Chemical structure and in silico docking of tiliroside to OLFM4.

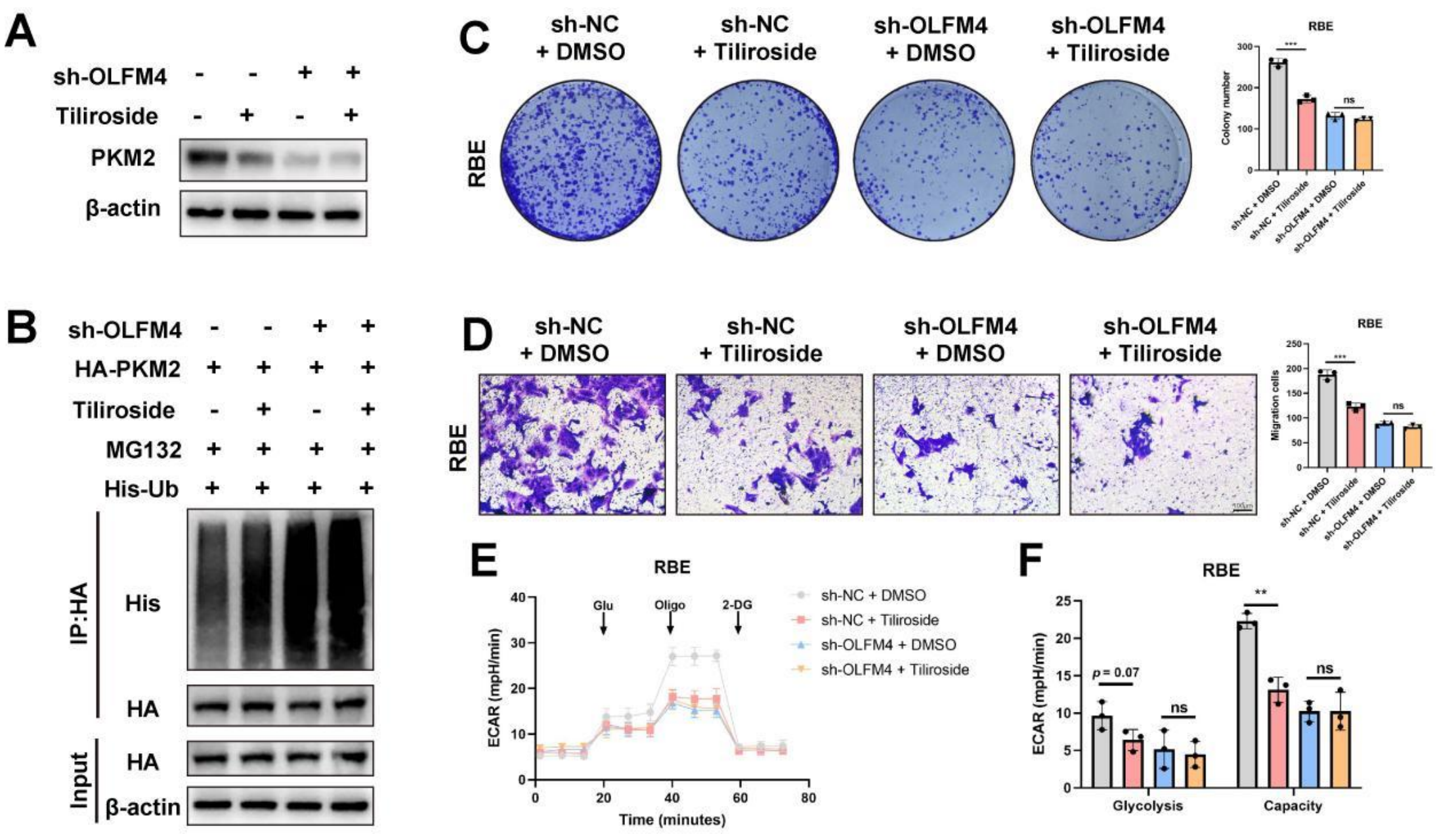

Figure S9 The inhibitory effects of tiliroside on ICC cells are dependent on OLFM4.

(A, B) Western blotting of PKM2 protein levels **(A)** and PKM2 ubiquitination assays **(B)** in RBE cells with or without OLFM4 knockdown following DMSO or tiliroside treatment. **(C)** Colony formation assays of RBE cells. **(D)** Transwell assays of RBE cells. Scale bar, 100 μm. **(E)** ECAR levels of RBE cells were measured. **(F)** Glycolysis rate and glycolytic capacity were analyzed. **P* < 0.05, ***P* < 0.01, ****P* < 0.001, ns, not significant.

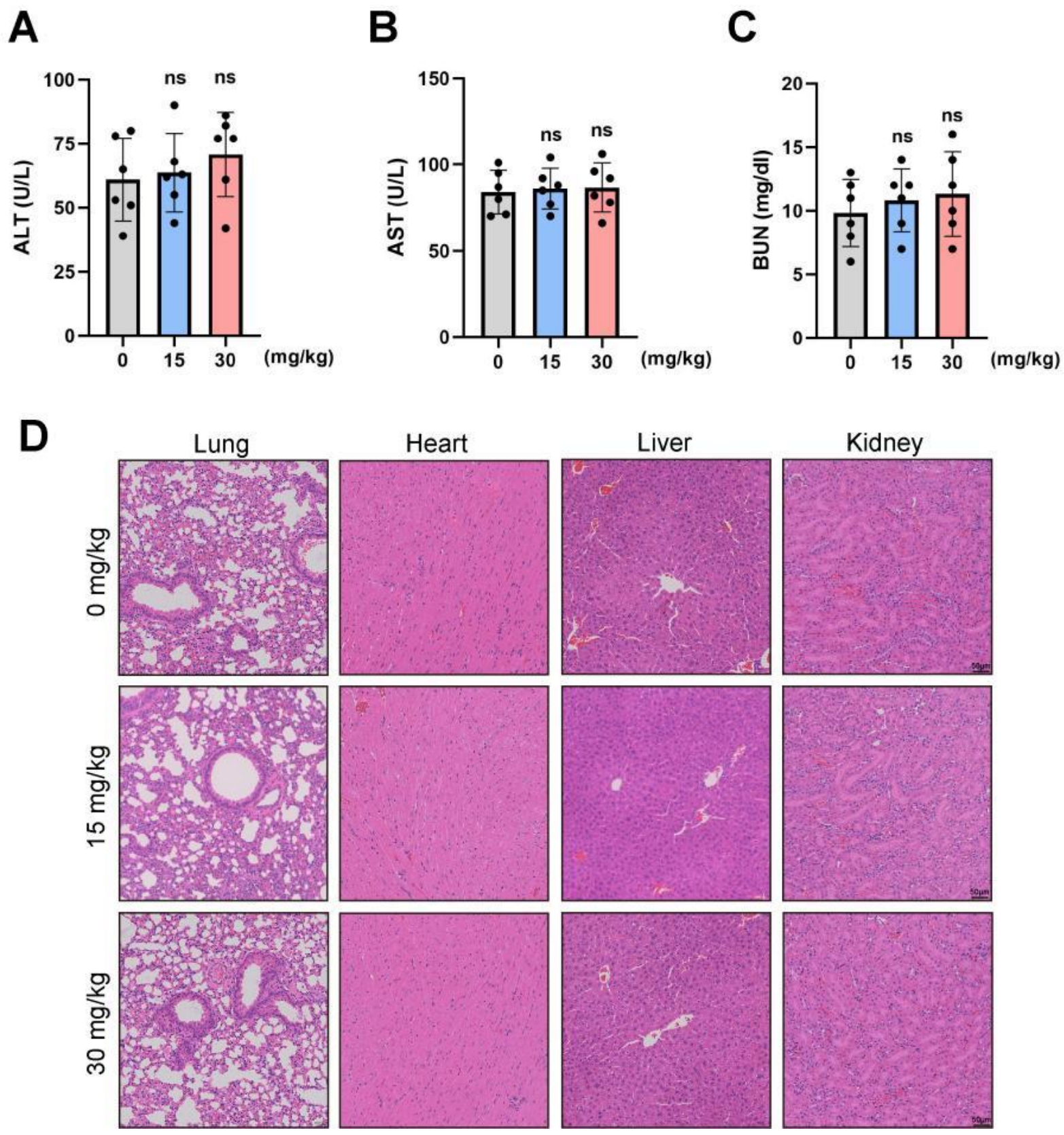

Figure S10 Evaluation of potential toxicity of tiliroside treatment.

(A-C) Serum levels of ALT (A), AST (B) and BUN (C) in mice treated with tiliroside at 0, 15, or 30 mg/kg. **(D)** Representative H&E staining of major organs showing no pathological abnormalities following tiliroside treatment. Scale bar, 50 μm. **P* < 0.05, ***P* < 0.01, ****P* < 0.001, ns, not significant.

Table S1 Relationship between OLFM4 and clinicopathological characteristics in Fu-iCCA cohort.

Variable	OLFM4 expression High (N = 103)	OLFM4 expression Low (N = 104)	*P* value
Sex			0.7328
Male	57	60	
Female	46	44	
Age			0.0725
<60	34	47	
≥60	69	57	
TNM stage			**< 0.001*** **
I-II	55	80	
III-IV	48	24	
HBsAg			0.0477*
Positive	22	35	
Negative	81	69	
Vascular invasion			0.5342
No	55	60	
Yes	48	44	
Intrahepatic metastasis			0.0699
No	62	75	
Yes	41	29	
Tumor size(cm)			0.5265
≤5	45	50	
>5	58	54	
Liver cirrhosis			0.5046
No	94	92	
Yes	9	12	

Regional lymph node metastasis			**< 0.001*** **
No	72	94	
Yes	31	10	
Distant metastasis			0.1894
No	96	101	
Yes	7	3	
Perineural invasion			0.1147
No	77	87	
Yes	26	17	
TB (μmol/L)			0.5996
<17.1	87	85	
≥17.1	16	19	
AFP (ng/mL)			0.6543
<20	94	93	
≥20	9	11	
CA19-9 (U/mL)			**< 0.001*** **
<37	32	62	
≥37	71	42	
CEA (μg/L)			**0.0075** **
<5	71	88	
≥5	32	16	
ALT (U/L)			0.3475
<40	82	88	
≥40	21	16	
γ-GT (U/L)			0.0579
<45	39	53	
≥45	64	51	

Table S2 Antibodies and reagents used in this study.

Name	Company	Cat no.
Anti-mouse OLFM4	Cell Signaling Technology	39141
Anti-human OLFM4	Cell Signaling Technology	14369
Anti- PKM2	Proteintech	60268-1-Ig
Anti-H3K18la	PTM Bio	PTM-1427RM
Anti-H3K9la	PTM Bio	PTM-1419RM
Anti-H4K5la	PTM Bio	PTM-1407RM
Anti-H4K8la	PTM Bio	PTM-1415RM
Anti-H4K12la	PTM Bio	PTM-1411RM
Anti-pan Kla	PTM Bio	PTM-1401
Anti- beta Actin	Proteintech	20536-1-AP
Anti-mouse IgG, HRP-linked Antibody	Cell Signaling Technology	7076
Anti-USP10	Abclonal	A4454
Anti-TRIM25	Proteintech	12573-1-AP
Anti-P300	lmmunoWay	YT5692
Anti-Flag	Sigma	F1804
Anti-HA	Cell Signaling Technology	3724
Anti-His	Proteintech	66005-1-lg
Anti-Histone H3	Cell Signaling Technology	9715
Anti-ubiquitin	Proteintech	10201-2-AP
Anti-Normal IgG	Cell Signaling Technology	2729
Anti-rabbit IgG, HRP-linked Antibody	Cell Signaling Technology	7074
2-DG	MedChemExpress	HY-13966
Lactic acid	MedChemExpress	HY-B2227
Oxamate	MedChemExpress	HY-W013032A
MG132	MedChemExpress	HY-13259
Cycloheximide	MedChemExpress	HY-12320

Diosmin	TargetMol	T0878
Schaftoside	TargetMol	T3898
Tiliroside	TargetMol	T5S1172
Indinavir sulfate	TargetMol	T5863

Table S3 Primers, siRNAs and probes used in this study.

Name	Sequence (5'-3')
Sh-NC	TTCTCCGAACGTGTCACGT
Sh-OLFM4-1	TGAGGGAATATGTCCAATTAA
Sh-OLFM4-2	GGAGGTGGAGATAAGAAATAT
Sh-OLFM4-3	ATGTACAACACCGGGAATATT
Si-PKM2	CTACCACTTGCAATTATTTGA
Si-P300	GAGGAGAGUAUACAUAUCUTT
OLFM4-F	ACTGTCCGAATTGACATCATGG
OLFM4-R	TTCTGAGCTTCCACCAAAACTC
beta-Actin-F	CAGCCTTCCTTCCTGGGCATG
beta-Actin-R	ATTGTGCTGGGTGCCAGGGCAG
